# Differential responsiveness of Holstein and Angus dermal fibroblasts to LPS challenge occurs without major differences in the methylome

**DOI:** 10.1186/s12864-016-2565-x

**Published:** 2016-03-24

**Authors:** Aimee L. Benjamin, Benjamin B. Green, Brian A. Crooker, Stephanie D. McKay, David E. Kerr

**Affiliations:** Department of Animal and Veterinary Sciences, University of Vermont, Terrill Hall, 570 Main Street, Burlington, VT 05405 USA; Geisel School of Medicine at Dartmouth, Department of Epidemiology and Department of Pharmacology and Toxicology, 7650 Remsen, Rope Ferry Rd, Hanover, NH 03755 USA; Department of Animal Science, University of Minnesota, Haecker Hall, 1364 Eckles Ave, St. Paul, MN 55108 USA

**Keywords:** Innate immunity, DNA methylation, RNA-Seq, MIRA-Seq

## Abstract

**Background:**

We have previously found substantial animal-to-animal and age-dependent variation in the response of Holstein fibroblast cultures challenged with LPS. To expand on this finding, fibroblast cultures were established from dairy (Holstein) and beef (Angus) cattle and challenged with LPS to examine breed-dependent differences in the innate immune response. Global gene expression was measured by RNA-Seq, while an epigenetic basis for expression differences was examined by methylated CpG island recovery assay sequencing (MIRA-Seq) analysis.

**Results:**

The Holstein breed displayed a more robust response to LPS than the Angus breed based on RNA-Seq analysis of cultures challenged with LPS for 0, 2, and 8 h. Several immune-associated genes were expressed at greater levels (FDR < 0.05) in Holstein cultures including *TLR4* at all time points and a number of pro-inflammatory genes such as *IL8, CCL20, CCL5*, and *TNF* following LPS exposure. Despite extensive breed differences in the transcriptome, MIRA-Seq unveiled relatively similar patterns of genome-wide DNA methylation between breeds, with an overall hypomethylation of gene promoters. However, by examining the genome in 3Kb windows, 49 regions of differential methylation were discovered between Holstein and Angus fibroblasts, and two of these regions fell within the promoter region (-2500 to +500 bp of the transcription start site) of the genes *NTRK2* and *ADAMTS5*.

**Conclusions:**

Fibroblasts isolated from Holstein cattle display a more robust response to LPS in comparison to cultures from Angus cattle. Different selection strategies and management practices exist between these two breeds that likely give rise to genetic and epigenetic factors contributing to the different immune response phenotypes.

**Electronic supplementary material:**

The online version of this article (doi:10.1186/s12864-016-2565-x) contains supplementary material, which is available to authorized users.

## Background

The innate immune response plays a critical role in pathogen detection and the resulting inflammatory response that arises to contain and eliminate foreign invasions into host tissues. Infection of the mammary gland resulting in mastitis is an important and well characterized disease of dairy cattle, such as the Holstein breed [1]. However, the incidence and severity of this disease in beef cattle has received little attention although two recent studies that examined mastitis prevalence in beef cows reported a moderate incidence with a surprising lack of *Escherichia coli* induced mastitis [2, 3]. This pathogen is a major cause of mastitis in dairy animals and thus differences in susceptibility to *E. coli* infection may exist between dairy and beef breeds. Bovine mammary infections due to *E. coli* typically cause an acute response with inflammation ranging from mild and quickly resolving, to severe forms that can lead to sepsis, shock, and even death [4]. The response is initiated following recognition of *E. coli* lipopolysaccharide (LPS) by the extracellular Toll-like receptor 4 (TLR4), which is expressed on multiple cell types within the mammary gland [5].

While there are multiple factors that can influence the inflammatory response to mammary gland infection, it has been suggested that host factors play the largest role in determining the severity of *E. coli* mastitis [6]. These host factors include age, stage of lactation, vaccination history, as well as genetic, and possibly epigenetic mechanisms all contributing to the magnitude and resolution of the host response. Identification of genetic or epigenetic factors controlling the inflammatory response may lead to enhanced selection or management strategies to produce dairy animals that have reduced collateral damage from mastitis. For example, genetic polymorphisms within pathogen recognition receptors or signaling pathway intermediates have been shown to lead to differences in disease susceptibility [7, 8]. Epigenetic mechanisms, such as DNA methylation or histone modifications can also influence gene expression resulting in phenotypic variation in immune response [9]. For example, human intestinal epithelial cells have increased DNA methylation in the *TLR4* gene promoter that reduces its expression and leads to a reduced responsiveness to LPS [10].

Selective breeding of cattle has been employed for many generations to produce breeds differing in economically important traits such as enhanced milk production in dairy breeds, and greater feed efficiency and meat quality in beef breeds [11, 12]. Cattle breeding has even resulted in sub-species (*Bos taurus* and *Bos indicus*) that differ markedly in resistance to disease such as that caused by tick infestation [13, 14] or challenge with *Theileria annulata* [15]. Results from these studies suggest that *B. taurus* animals develop a greater inflammatory response to pathogens than do breeds of the *B. indicus* subspecies. Within the *B. taurus* sub-breed, studies directly comparing the inflammatory response to mammary infection between dairy and beef breeds have not been conducted. To further investigate potential differences in immune responses between dairy and beef animals, we challenged dermal fibroblasts obtained from Holstein (dairy) and Angus (beef) cows with LPS and then used RNA-seq to examine global gene expression differences. We also employed MIRA-seq to examine if global differences in the methylome could explain differences in the transcriptome.

## Results

### Fibroblast IL-8 response

**Fig. 1:**
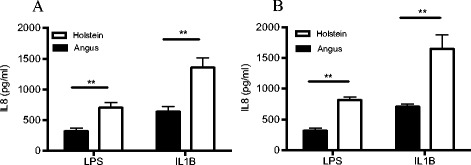
IL8 protein production from Holstein and Angus fibroblasts. **a** IL8 production by dermal fibroblasts isolated from 19-month old Holstein (*n* = 5) and Angus (*n* = 12) heifers and challenged for 24 h with LPS (100ng/ml) or IL1B (1ng/ml). **b** Eight cultures (*n* = 4/breed) were randomly chosen for RNA-seq and MIRA-seq analysis and levels of IL8 protein following a second 24 h challenge with either LPS (100ng/ml) or IL1B (1ng/ml) on these 8 cultures are shown. There was no detectable IL8 production from un-treated cultures of both breeds. Values are mean ± SEM. * indicates *P* < 0.05, ** indicates *P* < 0.01

Dermal fibroblasts (DF) isolated from Holstein and Angus heifers were used to investigate differences in the innate immune response between dairy and beef breeds. Fibroblasts isolated from Holsteins produced considerably higher levels of IL8 (*P* < 0.01) in response to a 24 h LPS (100ng/ml) challenge (Fig. 1a) when compared to Angus cultures (703 ± 86 pg/ml vs. 322 ± 46 pg/ml, respectively). A similar pattern was observed following IL1B (1ng/ml) treatment (Fig. 1a), with Holstein DF having a greater magnitude of IL8 response than Angus DF (1355 ± 160 pg/ml vs. 639 ± 80 pg/ml, respectively). There was no detectable IL8 in media collected from un-stimulated Holstein or Angus fibroblasts.

RNA-Seq and MIRA- Seq analysis was performed on eight cultures (*n* = 4/breed) randomly selected from the pool of animals. These cultures were revived simultaneously from cryopreservation and prepared for a second challenge with LPS and IL1B. The Holstein cultures produced approximately twice (*P* < 0.01) as much IL8 protein in response to LPS compared to the Angus cultures (Fig. 1b). Similarly, differential IL8 responses were observed following IL1B exposure, with Holstein cultures exhibiting a hyper-responsive phenotype in IL8 protein production (Fig. 1b).

### RNA-Seq analysis of Holstein and angus LPS response

Total RNA samples for RNA-Seq analysis were obtained from the four Holstein and four Angus cultures following LPS exposure for 0, 2, and 8 h. These 24 samples generated approximately 54 million reads per sample following quality control. Alignment to the UMD v3.1 bovine genome resulted in 95 % of the reads falling within alignment parameters (see Methods), thus an average of 51 million reads per sample. Under our definition of expression (CPM > 1 in at least 50 % of the samples), there were 20,356 targets detected at hour 0, which defined the core bovine fibroblast transcriptome under basal conditions. At hours 2 and 8 following LPS treatment, there were 21,411 and 21,590 targets detected, respectively. By combining breeds, and comparing basal gene expression to time points following LPS-induced innate immune response, 624 transcripts were revealed to have differential gene expression (FDR < 0.05; CPM > 1; FC ≥ 2) with 470 up- and 154 down-regulated at hour 2 post-LPS (Additional file 1). At hour 8 post-LPS, 331 transcripts were discovered having differential gene expression compared to hour 0, of which, 250 were up- and 81 were down- regulated (Additional file 2). LPS treatment of the fibroblast cultures induced various innate immune-associated genes in the LPS signaling pathway and those coding for response proteins (Table 1). There were temporal differences in the induction of these genes following LPS exposure for 2 or 8 h. Early induced genes included several pro-inflammatory chemokines and cytokines such *IL8, IL1A, TNF, chemokine (C-X-C) motif ligand* (*CXCL*) *2,* and *Interferon regulatory factors* (*IRF*) *1* and *5.* Following 8 h of LPS exposure, several Type I IFN-related genes showed greater expression compared to hour 0, while *TNF* expression had returned to basal levels.

**Table 1:** Average response to LPS from combined Holstein and Angus cultures measured by increases in expression of immune-associated genes compared to hour 0 post-LPS

Gene symbol	Gene Name	Fold Change^a^	
Transcription and activation pathways		Hour 2	Hour 8
BIRC3	Baculoviral IAP repeat-containing protein 3	7.3	4.2
NFKBIA	Nuclear factor of kappa light polypeptide gene enhancer in B-cells inhibitor, alpha	24.3	9.3
NFKBIZ	Nuclear factor of kappa-B inhibitor zeta	11.7	3.2
NFKB2	Nuclear factor of kappa light polypeptide gene enhancer in B-cells 2	- -	2.8
NFKBID	Nuclear factor of kappa light polypeptide gene enhancer in B-cells inhibitor, delta	2.3	- -
Cytokines, chemokines, and growth factors			
CCL2	Chemokine (C-C motif) ligand 2	14.9	14.6
CCL5	RANTES	49.6	132.8
CCL20	Chemokine (C-C motif) ligand 20	1102.1	3147.3
CXCL2	Chemokine (C-X-C motif) ligand 2	67.9	31.6
CXCL6	Chemokine (C-X-C motif) ligand 6	12.3	45.0
IL1A	Interleukin 1, alpha	9.2	5.7
IL6	Interleukin 6	88.2	124.1
IL8	Interleukin 8	325.7	387.8
SAA3	Serum Amyloid A 3	65.1	1145.6
TNF	Tumor Necrosis Factor, alpha	87.4	- -
Type I IFN-related genes			
IRF1	Interferon regulatory factor 1	12.3	4.2
IRF5	Interferon regulatory factor 5	3.0	- -
ISG15	Ubiquitin-like modifier	- -	308.3
MX2	Myxovirus (influenza virus) resistance 2	- -	156.6
OAS1	2'–5'- oligoadenylate synthetase 1	- -	128.7
OAS2	2'–5'- oligoadenylate synthetase 2	- -	65.6

^a^Data obtained by RNA-Seq and presented as fold induction of the indicated gene at either 2 or 8 h post-LPS in comparison to expression levels at 0 h post-LPS. All fold changes shown are FDR < 0.05; FC > 2; and CPM >1. - - indicates FDR > 0.05, FC < 2, or CPM < 1

**Fig. 2:**
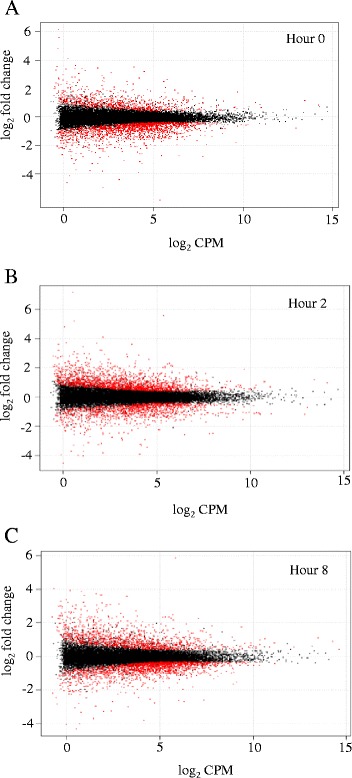
Scatter plots of RNA-Seq analysis. Eight fibroblast cultures (four of each breed) were used in RNA-Seq to explore gene expression differences. Scatter plots of indices analyzed from RNA-Seq data were generated for expression level (log_2_CPM) and differential expression (log_2_ Fold Change) at 0 h (**a**), 2 h (**b**), and 8 h (**c**) following LPS exposure. Positive fold change values indicate higher expression in Holstein cultures while negative fold change values show higher expression in Angus cultures. Red dots denote FDR < 0.05

A comparison between Holstein and Angus cultures revealed 844, 968, and 730 differentially expressed (DE) genes (FDR < 0.05; CPM > 1; FC ≥ 2) at hours 0, 2, and 8-post LPS, respectively (Fig. 2; Additional file 3). Of these genes, 369, 517, and 477, respectively, had higher expression levels in Holstein cultures compared to Angus cultures. Pathway enrichment analysis was completed with DAVID and genes with differential expression between the breeds at 2 h post-LPS were involved with the MAPK (*P* = 0.029) and TGF-beta (*P* = 0.031) signaling pathways. By 8 h post-LPS, the NOD-like (*P* = 0.003), Toll-like (*P* = 0.02), and RIG-I-like (*P* = 0.02) receptor signaling pathways were represented in our set of DE genes between the two breeds. Table 2 lists several of the DE genes that are involved in the innate immune response. *TLR4*, *NOD1*, and *TRAF1*, genes involved with the recognition of pathogen associated molecular patterns and activation of NFκB pathway, were more expressed in Holstein cultures. Chemokines such as *chemokine (C-C motif) ligand (CCL) 2*, *5*, and *20*, and *IL8* that recruit monocytes, T cells, lymphocytes, and neutrophils to sites of infection also had higher expression levels in Holstein fibroblasts. In addition to genes related to the innate immune response, differences were observed in genes that have been implicated in DNA methylation, such as the family of DNA methyltransferase (DNMT) genes and *ubiquitin-like with PHD and RING finger domains 1* (*UHRF1*) [16, 17]. Expression of *DNMT3A* and *3B*, which are involved with *de novo* DNA methylation, were similar between the breeds. However, expression of *DNMT1*, which is responsible for maintenance of DNA methylation patterns following DNA replication, was 1.6-fold higher at hour 0 and 1.5-fold higher at hours 2 and 8 in Angus cultures compared to Holstein cultures. Angus cultures also had 2.3–, 2.0-, and 2.0-fold higher expression of *UHRF1* at 0, 2, and 8 h post-LPS treatment (Table 2). Interestingly, greater activation of a potentially anti-inflammatory signaling pathway in Angus cultures was noted by the 3.1-fold higher expression of the gene encoding *chemokine (C-X-C motif) receptor 4 (CXCR4)* at hour 0 compared to Holsteins. While *CXCR4* expression was not different at hours 2 and 8 post-LPS, the sole ligand of this receptor, *CXCL12*, was expressed 5.7–, 7.4-, and 6.5-fold higher in Angus cultures at hour 0 and at 2 and 8 h post LPS stimulation.

**Table 2:** Differential gene expression (fold change) of immune-associated genes at hours 0, 2, and 8 post-LPS treatment of fibroblasts collected from Holstein animals as compared to fibroblasts collected from Angus animals

Gene	Hour 0	Hour 2	Hour 8
Innate immune related genes			
CCL2	- -	- -	2.9
CCL5	- -	- -	5.7
CCL20	- -	- -	8.6
CCL26	- -	2.8	3.6
CX3CL1	- -	- -	14.8
CXCL12	−5.7	−7.4	−6.5
CXCL16	- -	- -	2.3
CXCR4	−3.1	- -	- -
HMGB1	−1.9	−1.9	−1.8
IFIH1	- -	- -	3.6
IL1R1	- -	1.5	- -
IL8	- -	- -	4.5
IRF5	2.4	2.4	2.6
MX2	- -	- -	14.0
NOD1	- -	2.4	- -
TLR2	−3.5	−2.8	- -
TLR4	3.0	4.0	2.5
TLR6	1.8	- -	2.5
TNF	- -	6.9	- -
TNFSF18	6.5	6.0	5.1
TNFSF4	4.4	- -	2.6
TRAF1	2.4	2.4	- -
TRAIP	- -	−2.7	- -
DNA Methylation associated genes			
DNMT1	−1.6	−1.5	−1.5
DNMT3A	- -	- -	- -
DNMT3B	- -	- -	- -
UHRF1	−2.3	−2.0	−2.0

Data obtained by RNA-Seq and presented as fold change between Holstein and Angus cultures. Positive values indicate higher expression in Holstein cultures as compared to Angus cultures. All presented values indicate FDR < 0.05 with an average CPM > 1, while - - notes a values with either FDR > 0.05 or an average CPM < 1

### RT-qPCR confirmation of RNA-Seq

**Fig. 3:**
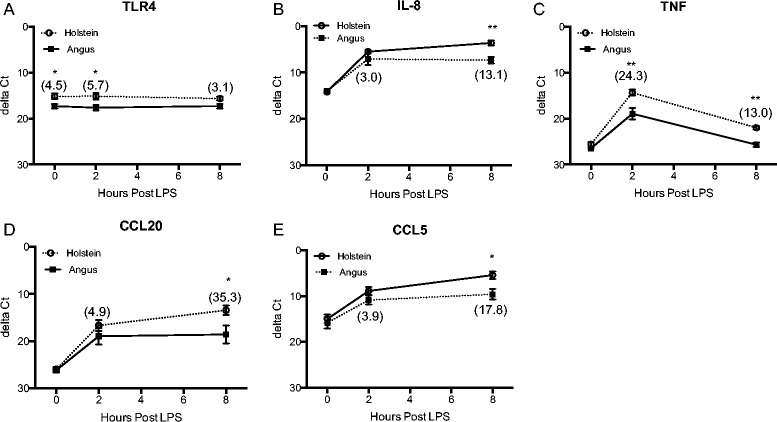
Selected gene expression by Holstein and Angus fibroblasts in response to LPS. Differences in expression of *TLR4* (**a**) *IL8* (**b**) *TNF* (**c**) *CCL20* (**d**) and *CCL5* (**e**) between Holstein and Angus fibroblast cultures at hours 0, 2, and 8 post-LPS exposure were determined by RT-qPCR. Values are expressed as delta Ct, or the difference in cycles to threshold (Ct) between the gene of interest and the endogenous gene control beta-actin (*ACTB)*. Fold differences (2^-ΔΔCt^) in expression between Holstein and Angus cultures are indicated in parentheses. All values are mean ± SEM. (*n* = 4/group). *P < 0.05 and **P < 0.01 (*t*-test)

Several immune-response associated genes were selected for RT-qPCR confirmation of expression differences observed between Holstein and Angus cultures following LPS treatment as determined by RNA-Seq. *TLR4*, *IL8*, *CCL20*, *CCL5*, and *TNF* showed similar expression levels in RT-qPCR analysis in comparison to the RNA-Seq data set. Basal expression of *IL8*, *CCL20*, *CCL5*, and *TNF* was similar between the breeds. However, by 2 h after LPS stimulation, marked differences were observed between Holstein and Angus expression of *IL8*, *TNF*, *CCL20*, and *CCL5*, with a 3.0–, 24.3-, 4.9-, and 3.9- fold higher expression in Holstein fibroblasts compared to Angus fibroblasts (Fig. 3 b, c, d, and e). A similar pattern was observed at 8 h, with Holstein cultures having higher expression in the following genes: *IL8* (13.1-fold), *TNF* (13.0-fold), *CCL20* (35.3-fold), and *CCL5* (17.8-fold). LPS exposure did not induce *TLR4* expression in either breed; however, Holstein cultures consistently expressed higher levels of *TLR4* compared to Angus cultures (Fig. 3a; 4.5–, 5.7-, and 3.1- fold higher at hours 0, 2, and 8, respectively). In general, gene expression values determined by RT-qPCR are in agreement with the transcriptomic results from RNA-Seq data.

### MIRA-Seq

The 8 DNA samples (4 per breed) processed for MIRA-Seq analysis resulted in approximately 44 million reads per sample following quality control. Alignment to the bovine UMD v3.1 reference genome yielded 97 % of reads falling within alignment parameters (see Methods) and an average of 43 million mapped reads per sample. To determine differential methylation rates between Holstein and Angus fibroblasts, the genome was divided into 3Kb windows, and then the read count in each window was compared. Analysis of the 3Kb windows revealed 49 regions that had different levels of methylation between Holstein and Angus cultures based on the following thresholds: FDR < 0.1; CPM > 1; FC ≥ 2 (Table 3). Of these differentially methylated regions (DMR), 24 had higher rates of methylation in cultures from Holstein animals, while 25 displayed higher methylation levels in Angus fibroblasts. Of the 24 regions with higher methylation in Holstein, 14 were found partially within an annotated gene while 10 were not. Within the 25 DMR with higher methylation levels in Angus cultures 13 fell within annotated genes while the remaining 12 regions did not. Only two of the DMR (greater methylation in Angus cultures) were located within the defined promoter regions of genes: *a disintegrin and metalloprotease with thrombospondin motifs 5* (*ADAMTS5*) and *neurotrophic tyrosine kinase, receptor type 2* (*NTRK2*). These DMRs were not reflected by differences in expression of either gene between the Holstein and Angus cultures at hours 0, 2, and 8 post-LPS (Table 3). A DMR spanning intron 3 and exon 4 of the *agouti signaling protein* (*ASIP*) gene had greater methylation in the Angus cells and was associated with a marked increase in its expression in these cells at all time points.

**Table 3:** Differentially methylated 3Kb regions between Holstein and Angus cultures as determined by MIRA-Seq and gene expression data for annotated genes

				RNA-Seq fold change^c^		
FC^a^	Chr	Genomic coordinates	Gene^b^	Hour 0	Hour 2	Hour 8
−2.9	1	8811701–8814700	**ADAMTS5**	- -	- -	- -
4.2	1	4686501–4689500	LOC100848874	- -	- -	- -
2.0	1	8325701–8328700	LOC526789	- -	- -	- -
−3.4	2	88463334–88466333	- -	- -	- -	- -
−3.4	2	88469334–88472333	- -	- -	- -	- -
2.6	2	18491734–18494733	OSBPL6	- -	1.4	1.5
2.0	5	116626706–116629705	FBLN1	- -	- -	- -
2.3	6	5529182–5532181	- - -	- -	- -	- -
−8.3	7	18532846–18535845	LOC100337044	- -	- -	- -
2.9	8	23178787–23181786	LOC100300143	- -	- -	- -
−2.6	8	34929387–34932386	- -	- -	- -	- -
−2.7	8	78669587–78672586	- -	- -	- -	- -
−2.8	8	12568687–12571686	- -	- -	- -	- -
−4.0	8	79335787–79338786	**NTRK2**	- -	- -	- -
−2.6	8	98801087–98804086	- -	- -	- -	- -
−2.6	9	20444051–20447050	- -	- -	- -	- -
−2.7	9	93571151–93574150	- -	- -	- -	- -
−4.0	9	88292251–88295250	ULBP1	- -	- -	- -
−2.7	10	32127701–32130700	- -	- -	- -	- -
2.1	12	51081922–51084921	- -	- -	- -	- -
−2.5	13	64236797–64239796	ASIP	−14.0	−15.2	−13.4
2.8	13	48576797–48579796	LRRN4	- -	- -	- -
2.6	14	36500947–36503946	- - -	- -	- -	- -
2.1	15	53581057–53584156	LOC100336675	- -	- -	- -
−2.3	15	9012857–9015856	- -	- -	- -	- -
−7.5	15	27535857–27538856	- -	- -	- -	- -
−2.8	15	55516557–55519556	SERPINH1	- -	- -	- -
−2.4	17	7224294–7227293	MAB21L2	- -	- -	- -
2.2	17	17977294–17980293	MAML3	- -	- -	- -
−2.8	18	60220698–60223697	LOC100848332	- -	- -	- -
−3.6	18	63217498–63220497	LOC783134	- -	- -	- -
3.4	18	33452798–33455797	- -	- -	- -	- -
2.4	19	42556475–42559474	- -	- -	- -	- -
2.3	19	62858675–62861674	AXIN2	- -	- -	- -
−2.4	19	43381475–43384474	CNTNAP1	- -	1.8	2.0
−3.6	19	23655275–23658274	HIC1	- -	1.7	- -
2.2	19	42109475–42112474	KRTAP9-2	- -	- -	- -
2.4	19	62705675–62708674	RGS9	2.0	2.1	2.5
4.8	23	49777993–49780992	- - -	- - -	- - -	- - -
2.8	24	59112931–59115930	CCBE1	−2.3	−2.2	−1.8
2.4	24	62388931–62391930	- - -	- - -	- - -	- - -
2.5	25	27266601–27269600	- - -	- - -	- - -	- - -
2.4	26	23658031–23661030	C26H10orf26	- - -	- - -	1.4
−2.4	26	16776031–16779030	LOC100848660	- - -	- - -	- - -
3.2	27	23720367–23723366	- - -	- - -	- - -	- - -
−2.4	X	63726195–63729194	LOC100848206	2.4	2.6	2.3
2.1	X	111529495–111532494	LOC516666	- - -	- - -	- - -
2.1	X	128195395–128198394	- - -	- - -	- - -	- - -
−2.6	X	55935095–55938094	- - -	- - -	- - -	- - -

^a^Data obtained by MIRA-Seq and presented as fold difference in read count of methylated regions. Positive fold change indicates higher methylation levels in Holstein cultures while negative values are higher methylation in Angus cultures

^b^DMRs with an associated gene indicate that some portion of the 3Kb region falls within an annotated gene, while - - indicates DMRs that are intergenic. Bolded gene names indicate that the discovered DMR fell within the promoter region of that gene (-2500 to +500bp from gene transcription start site)

^**c**^Positive values indicate greater expression in Holstein cultures as compared to Angus

### Influence of DNA methylation on gene expression

**Fig. 4:**
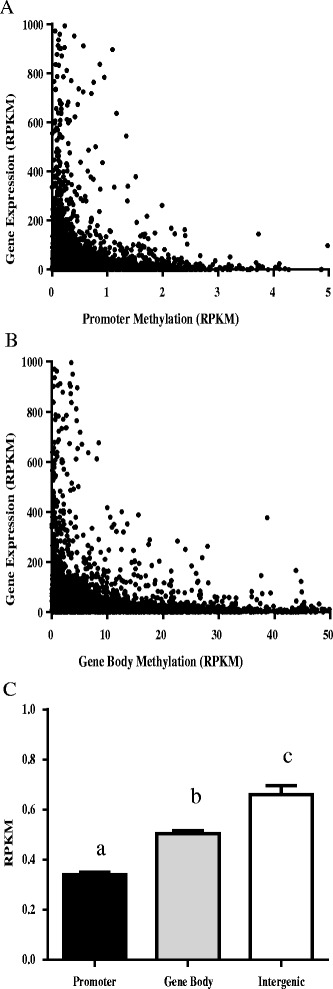
Role of DNA methylation on gene expression and differential methylation levels due to genomic location. As there were no significant effects of breed on the correlations between genomic location of DNA methylation and subsequent gene expression, data was combined for the 4 Holstein and 4 Angus cultures in this analysis. Scatter plots show the relation between gene expression at 8 h post-LPS treatment and levels of DNA methylation at the gene’s promoter (**a**) or gene body (**b**) Gene expression levels (RNA-Seq) and DNA methylation (MIRA-Seq) were normalized to gene size and presented as RPKM. **c** The average methylation levels of combined Holstein and Angus MIRA-Seq libraries based upon genomic region. Lettering denotes differential methylation levels as measured by a one-way ANOVA with Bonferonni post-test (*p* < 0.05). All values are mean ± SEM

The genome-wide association between gene expression and methylation levels within a gene body or gene promoter region was investigated by plotting MIRA-Seq values for a gene body or gene promoter against RNA-Seq values for 8 h post-LPS. As data from the two breeds appeared similar, the combined data points from both the Holstein and Angus cultures were binned as either low or high, with a gating value of reads per kilobase per million matched reads (RPKM) =5 for RNA-seq or RPKM = 0.5 for MIRA-Seq. When the relationship between methylation levels in either the gene promoter region or the gene body and that gene’s subsequent expression level was analyzed, it revealed the two values were significantly dependent upon one another (*P* < 0.001; O.R. = 0.83; 95 % C.I. = 0.76–0.89 and *P* < 0.001; O.R. = 1.26; 95 % C.I. = 1.14–1.40), for gene promoter (Fig. 4a) and gene body (Fig. 4b), respectively. This indicates a strong inverse relationship between methylation in either the gene promoter or gene body and the level of gene expression. Analysis of the 0- and 2-h time points following LPS treatment gave similar results (data not shown).

Levels of DNA methylation were also measured, as RPKM, in gene promoters, gene bodies, or intergenic regions (Fig. 4c). Data from un-treated cultures of the 4 Holstein and 4 Angus were combined for this analysis, as there were no significant breed differences. Levels of methylation in all three regions were significantly different (*P* < 0.05), with gene promoters having the lowest and intergenic regions having the highest levels of methylation.

## Discussion

Variation exists between cows in their innate immune response to mastitis. A greater understanding of the basis for this variation could facilitate new breeding, selection, or management practices to develop animals with superior ability to contain and eliminate pathogens from the normally sterile mammary gland. We have previously used dermal fibroblasts to model this animal variation within groups of Holstein cows [18, 19]. In these studies we found substantial differences between dairy cows in the magnitude of their fibroblasts’ response following treatment with LPS or PAM2CSK4 that are ligands for TLR4 and TLR2/6, respectively. Intra-mammary bacterial challenges were also conducted on groups of lactating animals from these studies that had been classified as having low or high responding phenotypes. Low responding animals developed a reduced inflammatory response characterized by less leakage of bovine serum albumin (BSA) into milk, as well as a less severe reduction in milk quality, as determined by a lower milk somatic cell count. This reduced response was accompanied by similar bacterial clearance rates between the phenotypes, suggesting the heightened response of the high responding animals did not offer any advantage in resolution of the infection. There is limited data to suggest that a high response phenotype is characteristic of dairy in comparison to beef breeds of cattle [13–15] and thus, in the current study, we have expanded our use of the fibroblast model to explore differences between a dairy breed (Holstein) and a beef breed (Angus) in their innate responses to LPS exposure.

Previous studies have looked at differences in the innate immune response between dairy breeds (Holstein and Jersey) following an intra-mammary challenge with *S. aureus* [20] or *E. coli* [21]. While there was high inter-animal variation in milk levels of IL8 and TNF during the challenge, the overall responses were similar between Holstein and Jersey animals. This suggested that the innate immune response is conserved between these two dairy breeds. Likewise, an intravenous LPS challenge comparing beef breeds (Angus and Romosinano) revealed small differences in the serum response profiles of TNF and IL1B [22]. Each of these studies examined differences within dairy or within beef breeds, while few studies have explored differences in innate immune responses between a dairy and a beef breed. In one such study, skin biopsies were collected from Holstein (*B. taurus*) and Brahman (*B. indicus*) animals following an experimental tick challenge to compare localized innate immune responses at tick-attachment sites [14]. Microarray analysis of the biopsies revealed that Holstein animals had greater expression of many innate immune response-associated genes in comparison to the Brahmans. It was suggested that the greater expression of chemokine and cytokines at tick-attachment sites might facilitate feeding by the tick, thereby leading to the lower resistance to tick infestations observed in the Holstein breed. Similarly, Holstein and Sahiwal (*B. indicus)* calves were used to explore breed differences in the inflammatory response [15]. In that study, calves from both breeds underwent an experimental challenge with *Theileria annulata,* the protozoan parasite that causes bovine theileriosis. Holstein calves experienced severe clinical symptoms following the challenge while Sahiwal calves seemed better able to control the inflammatory response. Furthermore, we have previously presented data from a related experiment, now being prepared for full publication, indicating that Holstein heifers have greater serum IL6 and TNF levels after an intravenous LPS challenge compared to age-matched Angus heifers [23]. In our current study, a comparison of Holstein and Angus fibroblasts revealed significant breed differences in response to an LPS challenge, with Holstein cultures exhibiting a hyper-responsive phenotype compared to the hypo-responsiveness of Angus cultures.

### Holstein vs. Angus differences in LPS response

RNA-Seq analysis of LPS-challenged fibroblasts revealed a large number of immune-associated differentially expressed genes between Holstein and Angus cultures. Many of these genes are known to play critical roles in the response to — and the recovery from — bacterial infections such as *E. coli* mastitis [6, 24]. In particular, *TLR4*, which is the extracellular receptor responsible for recognition of LPS, was expressed at higher levels in Holstein cultures before (4.5 fold) and following LPS stimulation (5.7 and 3.1 fold). This may suggest the Holstein animals will detect and respond to Gram-negative bacteria more vigorously than Angus animals. Additionally, several subunits of the NFKB transcription factor complex were differentially expressed between breeds. Specifically, Holstein cultures had greater expression of *RELB* (1.8-fold) at hours 0 and 2, and *NFKB1* (1.6-fold) and *NFKB2* (1.8-fold) at hour 8 post LPS treatment. This transcription factor is central to innate immune signaling pathways leading from a diverse array of pattern recognition receptors including the TLR and NOD receptor families [25]. Once activated, NFKB promotes the transcription of chemokine and cytokine genes associated with the innate immune system. While the activity of NFKB is carefully regulated under normal conditions, excessive production of pro-inflammatory cytokines can lead to its further activation which may create a chronic inflammatory condition [26]. The higher expression of both TLR4 and the various subunits of NFKB complex in the Holstein fibroblasts would enable the greater responsiveness to LPS observed in this breed.

An appropriate innate immune response is also dependent on timely production of anti-inflammatory factors once the stimulatory agent has been eliminated. The chemokine receptor 4 (CXCR4) and its sole ligand, CXCL12 are important in this regard and their interaction has been shown to suppress TLR4 signaling and NFKB activation in response to LPS in human embryonic kidney cells [27] and to suppress LPS-induced IL6 production by murine bone marrow-derived macrophages [28]. The CXCL12 chemokine has the ability to activate anti-inflammatory pathways in T-cells and macrophages and to suppress inflammation [29, 30]. A single injection of a CXCL12 analog administered to mice in parallel with LPS was able to decrease the levels of LPS-induced plasma TNF [28]. Within our fibroblasts, Angus cultures expressed greater levels of *CXCR4* and *CXCL12* prior to LPS treatment. At hours 2 and 8 post-LPS, *CXCL12* expression continued to be higher in Angus while the differences in *CXCR4* expression disappeared. Due to the inhibitory function of CXCR4 and CXCL12, the higher basal expression of these two genes in Angus cultures may have reduced their LPS responsiveness.

Breed differences in the expression of genes associated with the maintenance of DNA methylation were also uncovered in RNA-Seq analysis. *DNMT1* and *UHRF1* were more highly expressed in Angus cultures both basally and following LPS exposure. DNMT1 is responsible for maintaining DNA methylation patterns following replication [16], and UHRF1 interacts with hemimethylated DNA and recruits DNMT1 to facilitate methylation of the daughter strand [17]. Greater expression of these two genes in Angus cultures may indicate this breed is better able to maintain methylation levels. Increased methylation of gene promoter regions is generally associated with reduced gene transcription and thus could account for the reduced expression of pro-inflammatory genes in fibroblasts from this breed.

### Methylation differences between breeds

The breed comparison also included an examination into the potential role that differences in early life environment might play in determining breed differences in an animal’s epigenome. Holstein calves are removed from their dams within 24 h of birth, moved to individual housing, and fed a milk replacer diet; while Angus calves are allowed to nurse from the dam for months and are typically housed with the herd out on pasture. These environmental differences could affect DNA-methylation patterns and lead to differences in gene transcription in response to immune stimulation. The MIRA-Seq analysis of Holstein and Angus fibroblasts revealed 49 3Kb regions of the genome that were differentially methylated between breeds (Table 3). In a global sense, the breed difference was minimal despite the higher expression of *DNMT1* and *UHRF1* in Angus cultures. Unexpectedly, genes involved in the LPS-response pathway were under-represented in these 49 regions. While it would appear that DNA methylation does not play a major role in the differential breed response to LPS observed in our fibroblast model, MIRA-Seq is only a moderate-resolution technique and a potential limitation of the current study. The MIRA technique is based on the high affinity of a two-protein complex formed by methyl-CpG-binding proteins (MBD) 2b and 3L1, which bind to methylated DNA in a methylated CpG density-dependent manner [31]. Regions of fragmented DNA with greater methylated CpG content, such as CpG islands, will have enhanced affinity for the MBD complex, allowing for enrichment of these regions in the final analysis, whereas regions with low CpG content will be under-represented. While this allows for a comparison of the overall methylome between individuals, MIRA-Seq is not sensitive enough to detect more subtle, but influential, differences in methylation at specific CpG sites. For example, differential methylation at a single CpG site in the *IL6* gene promoter was attributed to differential serum levels of IL6 in patients suffering from rheumatoid arthritis [32]. Patients that were experiencing elevated serum levels of IL6 had lower methylation rates at a single CpG site in the gene promoter, suggesting that methylation at this site represses *IL6* transcription. Subtle differences in CpG methylation between Holstein and Angus cultures may have been lost in our analysis. Future studies of methylation differences between cattle breeds would benefit from a technology with greater resolving power such as those based on sequencing of bisulfite converted DNA [33].

### Identification of biomarkers linked to phenotypic responses

Previously, we have demonstrated age-dependent increases in LPS-responsiveness of fibroblasts collected from the same animals at 5 and 16 months of age [34]. Cultures isolated from young (5-month-old) Holsteins produced considerably lower levels of IL8 following LPS exposure compared to cultures collected from the older (16-month-old) Holsteins. RNA-Seq analysis was completed on the young vs. old cultures following LPS treatment, and revealed several pro-inflammatory response genes, including *IL8*, *IL6*, *CCL20*, *TNF*, and *CXCL2* that had greater expression in older cultures following LPS stimulation [35]. Comparing the RNA-Seq data sets from the previous young vs. older Holstein study, and the current Holstein vs. Angus study revealed several genes that had similar expression patterns defining a high vs. low response phenotype. The higher response phenotype of older animals in the previous study, and Holstein animals in the current study, is characterized by greater expression of *TLR4, IL8, CCL2, CCL5, CCL20*, and *TNF.* These genes are critical in the recognition of and resulting inflammation following infection by Gram-negative bacteria. The identification of this set of biomarkers from the global transcriptome may allow for greater precision in classifying an animal’s innate immune response phenotype even at a young age. This has potential utility in selecting low response replacement heifers as young calves to avoid raising high response animals that are more likely to develop severe rather than milder forms of coliform mastitis. Alternatively, selective modification of the Holstein genome with appropriate Angus genes may be used to generate Holstein animals with a reduced, potentially beneficial, innate response to infection.

## Conclusions

Our previous work on characterizing within-breed variation in innate immune response has now been extended to reveal substantial differences between a dairy and a beef breed. Our fibroblast model system indicates that divergent selection for dairy or beef character has resulted in substantial differences in immune phenotypes between these breeds, with dairy animals being more responsive to LPS than beef animals. Genetic differences between the dairy and beef breeds are likely of key importance, but management differences resulting in differing *in utero* and neonatal environments may lead to epigenetic differences as well. Events such as health of the dam during pregnancy or the level of maternal care a calf receives in early life may lead to epigenetic modifications that alter that animal’s innate immune response phenotype as an adult. Our current studies also provide a set of gene expression biomarkers, measurable in dermal fibroblasts, which are indicative of an animal’s innate immune response magnitude.

## Methods

### Animals and experimental set-up

Female animals of two cattle breeds were used in this study: Angus (*n* = 12) and Holstein (*n* = 5). All animals were 19.4 (±0.1) months of age at the time of skin sampling (see below) and were housed and cared for at the University of Minnesota. The Institutional Animal Care and Use Committee at both the University of Vermont and the University of Minnesota approved all animal procedures prior to beginning the study.

### Dermal fibroblast isolation

Skin biopsies were collected from the shoulder area of animals housed at the University of Minnesota as previously described [18] and shipped overnight on ice packs to the University of Vermont in a transport media consisting of 1X Dulbecco’s PBS (DPBS; Hyclone Laboratories, Logan, UT) with 1X antibiotic cocktail (100 U/mL penicillin, 100μg/mL streptomycin, and 0.25μg/mL amphotericin B; Hyclone Laboratories). Once received, biopsy samples were processed as described [19]. Briefly, fibroblasts were isolated with a 0.5 % collagenase type I enzyme solution (Life Technologies, Grand Island, NY) and seeded in a 25cm^2^ flask (Corning Inc., Corning, NY) in Dulbecco’s Modified Eagle Medium (DMEM; Hyclone Laboratories) with 10 % FBS (Hyclone Laboratories), 1X antibiotic cocktail, and 1X Insulin-Transferrin-Selenium (ITS; Mediatech Inc., Herndon, VA). Upon confluency, cells were detached with 0.25 % trypsin (MP Biomedical, Santa Ana, CA) and seeded into a 75cm^2^ flask (Corning Inc.) in DMEM with 5 % FBS, 1X antibiotic cocktail, and 1X ITS. After approximately four days, cells were expanded into three 75cm^2^ flasks, and once confluent, cells were detached from the flasks with trypsin, diluted in DMEM supplemented with 20 % FBS and 10 % dimethyl sulfoxide (Sigma- Aldrich, St. Louis, MO), and nine aliquots of third passage cells were cryopreserved in liquid nitrogen for subsequent challenges.

### In vitro challenges

A challenge was conducted to compare breed differences in the fibroblast response to LPS exposure on fibroblasts isolated from Holstein and Angus animals. Aliquots of fibroblasts isolated from both breeds were revived in parallel from cryopreservation and cultured in a 75cm^2^ flask in DMEM supplemented with 5 % FBS, 1X antibiotic cocktail, and 1X ITS. Once confluency was reached, cells were detached with 0.25 % trypsin, washed, counted with a cell counter (Bio Rad, Hercules, CA) and seeded into 6-well plates (Corning Inc.) at 1.25 × 10^5^ cells/ml in a total volume of 2ml. Media was replaced 24 h later with 2ml of either fresh media (negative control), media containing 100ng/ml of ultra-pure LPS isolated from *Escherichia coli* O111:B4 (Sigma-Aldrich), or media containing 1ng/ml of recombinant bovine IL1B (AbD Serotech, Raleigh, NC). After the challenge period, media was collected from each well, spun at 10,000x *g* for one minute to remove cell debris, and stored at -20 °C until further analysis.

### IL8 ELISA

The concentration of IL8 in conditioned media samples was determined by a custom sandwich ELISA as described previously [24]. Mouse anti-bovine (clone 170.13, gifted by Samuel Maheswaren, University of Minnesota, St. Paul, MN) and a biotinylated goat anti-human IL8 (R&D Systems Inc., Minneapolis, MN) were used as capture and detection antibodies, respectively. Recombinant bovine IL8 (Thermo Scientific, Rockford, IL) was used as the assay standard. The detection limit for this assay was 130pg/ml. Differences in IL8 protein production between Holstein and Angus fibroblast cultures were determined using a Student’s *t*-test (Graph Pad Prism 6.0).

### RNA-Seq

Fibroblast cultures collected from four animals of each breed (Holstein and Angus) were randomly chosen for investigation of whole transcriptome (RNA-Seq) differences between the breeds. Aliquots from each culture were revived from cryopreservation and grown to confluency in a 75cm^2^ flask with DMEM supplemented with 5 % FBS, 1X antibiotic cocktail, and 1X ITS. Cells were lifted with 0.25 % trypsin, counted, and seeded into 6-well plates at 1.00 × 10^5^ cells/ml. Following a 48 h incubation, fibroblasts were challenged with 100ng/ml of LPS and RNA was collected from replicate wells at three time points: hour zero (control conditions), hour two, and hour eight post-LPS using the PurefectPure RNA Cultured Cell extraction kit (5 Prime, Hamburg, Germany), which includes a DNase treatment step to eliminate DNA contamination. RNA concentration and quality were assessed using a Qubit Spectrofluorometer (Life Technologies, Carlsbad, CA) and an Agilent Bioanalyzer 2100 (Agilent Technologies, Santa Clara, CA), ensuring that all samples had an RNA integrity number (RIN) of 9.5 or greater. Libraries for RNA-seq analysis were constructed as previously described [35]. Briefly, 500ng of total RNA was PolyA enriched using magnetic beads, reversed transcribed, and the resulting cDNA was fragmented, end repaired, and adenylated. Oligonucleotide adaptors (Illumina, San Diego, CA), each with a unique adaptor sequence or barcode, were then ligated onto each sample. PCR amplification was completed using Illumina reagents, followed by quality assessment (as described above), and high accuracy qPCR quantification (KAPA Biosciences kit # 4824, Barre, VT). AMPure XP Magnetic Beads (Beckman Coulter, Pasadena, CA) were used in cDNA clean-up steps. Sequencing was performed using a 12pM/flow cell lane on an Illumina CBOT for flow cell cluster generation and the Illumina HiSeq1000 for sequencing by synthesis equipped with the HiSeq Control and sequence analysis software.

### DNA isolation and methylated CpG island recovery assay (MIRA-Seq)

MIRA-Seq libraries were generated to investigate genome wide methylation levels in fibroblasts isolated from Holstein and Angus cattle. Genomic DNA was isolated from the same Holstein (*n* = 4) and Angus (*n* = 4) cultures used in the RNA-Seq experiment (with no exposure to LPS) using the 5 Prime Pure Perfect Archive DNA Extraction kit (Hamburg, Germany). Following extraction, genomic DNA was sonicated and processed into MIRA-seq libraries of approximately 300–700 bp in size, essentially as described previously [35] starting with 1.5μg of DNA. MIRA pull-down was performed using the Methyl Collector Ultra Kit (Active Motif, Carlsbad CA) per manufacturer’s instructions. Sequencing of the libraries was performed using a 12pM/lane bridge amplification on an Illumina CBOT for flow cell cluster generation and the HiSeq1000 for sequencing by synthesis equipped with the HiSeq Control and sequence analysis software.

### Analysis of RNA-Seq data

Raw sequence reads that had a median quality (Q) score of less than 20, more than 3 uncalled bases, or were less than 25bp following trimming were removed from further analysis, and filtered reads were aligned to the reference UMD v3.1 [36] bovine genome using the software package NextGENe v. 2.3.4 (Softgenetics, State College, PA). In order for a read to be considered a mapped read, alignment parameters required  > 85 % of the read’s length to align to the reference sequence. After reads were mapped using NextGENe, total raw read counts were generated for each annotated gene, as defined by the UCSC genome browser UMD v 3.1/bosTau 6.

RNA-Seq data was analyzed by statistical methods used by edgeR in the R software package (version 3.0.1). Initially, genes with a low read count, defined as at least one mapped read per million mapped reads (counts per million; CPM) in less than 50 % of the samples being compared, were eliminated. For example, comparison of cultures from Holstein and Angus animals at a given time point required analysis of *n* = 4 samples/breed, so at least 4 samples needed a CPM equal to or greater than 1 to be considered for analysis.

A generalized linear model likelihood-ratio test using the limma package was used to conduct comparisons of cultures from animals of the two breeds at the different time points post-LPS for RNA-Seq. Similar analysis was also employed to determine the LPS response (0 h vs. 2 h and 0 h vs. 8 h post-LPS). Raw *p*-values were adjusted to account for multiple comparisons using the Benjamini-Hochberg method [37].

When analyzing the effect of breed on the LPS response, genes were considered differentially expressed if they passed the false discovery rate (FDR) < 0.05 and fold change (FC) ≥ 2 thresholds. The effect of LPS on gene expression was determined by comparing cultures at the 2- and 8-h time points to the 0 h cultures. The Database for Annotation, Visualization and Integrated Discovery (DAVID; http://david.abcc.ncifcrf.gov/) [38, 39] was used for functional annotation and analysis by uploading the official gene symbol of statistically significant genes (FC ≥ 2; CPM > 1; FDR < 0.05 for RNA-Seq).

### Analysis of MIRA-Seq

Raw sequence reads were filtered and aligned as described above. After reads were mapped using NextGENe, total raw read counts for each gene, as defined by the UCSC genome browser, UMD v 3.1/bosTau6, were generated. Data analysis was completed using the edgeR module in the R software package (v3.0.1). Genes with a low read count, defined as a CPM of at least 1 in less than 50 % of the samples were removed. A generalized linear model likelihood-ratio test using the limma package was employed to compare methylation levels between cultures from Holstein and Angus animals. Identification of differentially methylated regions (DMR) was completed on 3Kb windows of the genome to allow for an overall assessment of the bovine fibroblast methylome. Raw *p*-values were adjusted to account for multiple comparisons using the Benjamini-Hochberg method [37]. For analysis of MIRA-Seq data, levels of methylation were considered different between breeds if the region passed the FDR < 0.1 and FC ≥ 2 thresholds.

### Quantitative real-time PCR

Five immune-associated genes from those identified in RNA-Seq were selected for quantitative real-time PCR (RT-qPCR). Oligonucleotide primers specific for the following genes were used: *Toll-like receptor 4* (*TLR4*), *interleukin 8 (IL8)*, *tumor necrosis factor alpha* (*TNF*), *chemokine (C-C-motif) ligand 5* (*CCL5*), and *chemokine (C-C-motif) ligand 20* (*CCL20*). Primer sequences are in Table 4. The RNA that was used for the whole transcriptome analysis was also used in RT-qPCR. The Improm II Reverse Transcriptase kit (Promega, Madison, WI) was used to complete first strand cDNA synthesis. Gene expression was quantified by RT-qPCR on a CFX96 Real-Time Instrument (Bio-Rad) using PereCTa SYBR Green Super-Mix, Low ROX kit (Quanta Biosciences). Cycling conditions were: initial denaturation at 95 °C for 2 min; then 40 cycles of denaturation at 95 °C for 15 s, annealing at 60 °C for 30 s, and extension at 72 °C for 1 min. Samples were run in duplicate and melt curves were performed to check amplification of desired gene product. The *beta-actin* (*ACTB*) gene was used as a reference gene for normalization [40, 41]. Cycles to threshold (Ct) were calculated for each sample and analyzed with the ΔCt method with fold change being 2^-ΔΔCt^. Analysis of RT-qPCR data was completed using a two-way ANOVA model with repeated measures (Graph Pad Prism 6.0). Comparisons with *P* < 0.05 were considered statistically significant within experiments.

**Table 4:** RT-qPCR primer pairs used for amplification of target genes

Gene	Forward Primer Sequence	Reverse Primer Sequence	Reference
TLR4	ACTGCAGCTTCAACCGTATC	TAAAGGCTCTGCACACATCA	[42]
IL8	GCTGGCTGTTGCTCTCTTG	AGGTGTGGAATGTGTTTTTATGC	[43]
TNF	TCTTCTCAAGCCTCAAGTAA	CCATGAGGGCATTGGCATAC	[44]
CCL20	TTCGACTGCTGTCTCCGATA	GCACAACTTGTTTCACCCACT	[5]
CCL5	CTGCCTTCGCTGTCCTCCTGATG	TTCTCTGGGTTGGCGCACACCTG	[5]
ACTB	GCAAATGCTTCTAGGCGGACT	CAATCTCATCTCGTTTTCTGCG	[43]

### Relationship between MIRA-Seq and RNA-Seq

To determine a relationship between DNA methylation and gene expression, the average reads per kilobase per million matched reads (RPKM) from the RNA-Seq and MIRA-Seq of the 8 cultures from the two cattle breeds were investigated. Gene body and intergenic regions were determined based on annotations of the UMD v3.1 bovine genome, while gene promoters were defined as -2500 to +500 bp from the gene transcription start site. A two-tailed Fisher’s exact test in the R software package was used to determine the relationship between mRNA transcription levels and DNA methylation, in which, an association was investigated between low or high levels of methylation and either low or high gene expression. Values for gene expression RPKM were calculated as the cumulative size of the gene exons, while for gene methylation, gene body length was the total size of both intronic and exonic segments. All values were normalized to library and transcript size by conversion of read counts into RPKM values. RNA-Seq RPKM values were binned into either low or high levels at a cutoff of RPKM = 5, while MIRA-Seq RPKM values were divided into low and high levels at PRKM = 0.5.

To determine if the type of genomic region had an effect on DNA methylation levels, average RPKM was calculated for gene promoters, gene bodies, and intergenic regions. Gene body and intergenic regions were determined by UMD v3.1 bovine genome, and gene promoters were defined as -2500 to +500 bp of a gene transcription start site. A one-way ANOVA with a Bonferroni post-test for multiple comparisons was run to determine differential methylation levels based on genomic location.

## Availability of supporting data

The datasets supporting the conclusions of this article are available in NCBI’s Gene Expression Omnibus and are accessible through GEO SuperSeries accession number GSE72075 https://www.ncbi.nlm.nih.gov/geo/query/acc.cgi?acc=GSE72075.

## Additional files

Additional file 1: Genes displaying differential gene expression (FDR < 0.05; CPM > 1; 2 ≤ FC ≤ -2) due to LPS at hour 2 as compared to hour 0. A positive fold change indicates higher expression at hour 2 compared to hour 0. CPM = counts per million. FDR = false discovery rate. Data shown for comparisons with an FDR < 0.05; CPM > 1; FC ≥ 2. (PDF 77 kb) Additional file 2: Genes displaying differential gene expression (FDR < 0.05; CPM > 1; 2 ≤ FC ≤ -2) due to LPS at hour 8 as compared to hour 0. A positive fold change indicates higher expression at hour 8 compared to hour 0. CPM = counts per million. FDR = false discovery rate. Data shown for comparisons with an FDR < 0.05; CPM > 1; FC ≥ 2. (PDF 63 kb) Additional file 3: Differentially expressed genes (FDR < 0.05; CPM > 1; 2 ≤ FC ≤ -2) between Holstein and Angus fibroblast cultures exposed to 100ng/ml LPS for 0, 2, and 8 h. A positive fold change indicates higher expression in Holstein cultures. CPM = counts per million. FDR = false discovery rate. (PDF 207 kb)
