# Supplementary material for: Differential responsiveness of Holstein and Angus dermal fibroblasts to LPS challenge occurs without major differences in the methylome
Source: BMC Genomics. 2016 Mar 24;17:258. doi: 10.1186/s12864-016-2565-x (PMC4806443; doi:10.1186/s12864-016-2565-x)
Supplement: Additional file 1: — Genes displaying differential gene expression (FDR < 0.05; CPM > 1; 2 ≤ FC ≤ -2) due to LPS at hour 2 as compared to hour 0. A positive fold change indicates higher expression at hour 2 compared to hour 0. CPM = counts per million. FDR = false discovery rate. Data shown for comparisons with an FDR < 0.05; CPM > 1; FC ≥ 2. (PDF 77 kb) [file 12864_2016_2565_MOESM1_ESM.pdf]

Gene	Chromosome	FC	CPM	FDR
CX3CL1	18	994.64	2.29	9.47E-29
IL8	6	325.72	21.20	1.03E-61
CD83	23	117.68	1.86	3.58E-41
IL6	4	88.16	21.40	2.79E-22
FOSB	18	86.78	29.47	3.15E-56
ARC	14	71.60	1.33	2.39E-36
RND1	5	69.59	29.82	4.85E-14
CXCL2	6	67.91	77.53	4.95E-61
XIRP1	22	53.97	17.27	1.96E-22
EGR3	8	51.35	5.99	5.31E-33
CCL5	19	49.57	9.24	2.20E-17
SLCO4A1	13	48.94	4.68	1.61E-20
TNFAIP3	9	43.53	15.77	1.03E-41
ZBTB46	13	42.37	1.38	3.59E-43
C2CD4B	10	39.43	1.11	2.45E-14
PTX3	1	33.82	6.15	1.76E-11
PTGS2	16	28.68	116.12	9.12E-43
LOC100138376	16	28.19	72.70	1.53E-42
LIF	17	24.90	31.21	9.87E-15
NFKBIA	21	24.25	72.14	3.11E-46
CTSW	29	18.81	6.58	2.05E-20
HAS2	14	18.81	7.02	1.07E-22
CCL2	19	14.88	80.20	1.08E-16
RGS16	16	14.63	17.51	3.29E-38
CFB	23	14.52	6.03	2.94E-07
F3	3	13.49	142.24	2.30E-07
DUSP5	26	12.53	6.63	1.61E-20
IRF1	7	12.31	24.94	2.22E-22
LOC100848038	7	12.28	54.97	2.17E-23
CXCL6	6	12.27	70.53	2.74E-13
NFKBIZ	1	11.73	52.37	4.20E-28
VCAM1	3	10.70	1.96	8.06E-09
KCNN4	18	9.96	1.59	2.72E-12
NFATC1	24	9.88	80.21	1.69E-50
CDA	2	9.46	1.20	3.10E-10
IL1A	11	9.24	1.50	1.63E-08
HBEGF	7	8.47	2.58	7.36E-18
LOC100337435	21	8.42	21.77	6.19E-08
WNT10B	5	7.44	7.89	1.25E-19
NR4A1	5	7.31	59.10	4.94E-30
BIRC3	15	7.28	45.80	7.79E-21
LOC518986	19	7.10	2.58	6.01E-07
GCNT4	10	7.05	3.99	2.24E-16

IER3	23	6.47	85.23	2.08E-23
ZC3H12A	3	6.34	19.90	1.11E-18
LOC100336748	9	6.07	1.28	0.0207
IL11	18	5.96	1.27	1.66E-16
SAMD11	16	5.86	42.06	6.26E-18
NR4A3	8	5.57	13.06	1.50E-26
LOC782264	12	5.41	3.10	2.70E-15
MAP3K8	13	5.24	6.70	2.65E-08
HAS1	18	5.19	1.61	6.32E-07
TMEM88	19	5.15	1.43	9.34E-07
MARCKSL1	2	5.00	10.21	8.71E-17
N4BP3	7	4.98	6.13	7.67E-19
RELB	18	4.84	19.95	2.67E-19
PHF21B	5	4.82	6.00	1.94E-22
PIM1	23	4.81	26.93	3.01E-14
JAG2	21	4.70	1.17	8.42E-05
ELFN1	25	4.60	2.23	1.24E-11
CCDC85B	29	4.54	29.59	2.63E-11
NAB2	5	4.52	63.42	7.32E-77
ERRFI1	16	4.37	124.07	8.54E-17
FOSL1	29	4.35	89.25	4.90E-08
LOC100847310	18	4.26	172.82	5.56E-34
NFATC2	13	4.19	2.71	2.35E-08
OLR1	5	4.18	41.72	7.26E-05
PLAUR	18	4.17	156.18	5.36E-34
TNFRSF1B	16	3.99	3.10	4.05E-17
TRIB1	14	3.97	19.12	5.08E-16
HIVEP3	3	3.97	4.98	1.45E-14
CILP2	7	3.96	2.53	6.04E-13
GPR68	21	3.94	1.35	0.000212
ZSWIM4	7	3.93	14.21	4.59E-13
JUNB	7	3.91	66.80	1.76E-12
SMAD7	24	3.91	24.56	7.59E-25
SERPINB2	24	3.82	6.52	0.00341
IL15RA	13	3.74	21.72	5.51E-31
KDM6B	19	3.72	48.60	5.03E-09
CD37	18	3.70	3.13	5.34E-15
LOC100847884	4	3.68	3.60	9.73E-15
TMEM100	19	3.67	1.07	0.00273
LOC100336535	19	3.65	1.58	0.0124
PDE4B	3	3.64	8.30	4.28E-07
NFKB1	6	3.58	31.22	6.27E-22
LOC509420	8	3.53	1.90	2.36E-12
NPAS4	29	3.51	2.38	6.76E-11
C13H20orf112	13	3.50	5.03	7.07E-10

ATP8A2	12	3.49	6.91	1.82E-17
GPC6	12	3.48	21.95	3.17E-08
DNAH2	19	3.47	1.68	2.44E-06
NUAK2	16	3.43	30.05	4.30E-17
DUSP6	5	3.42	12.79	1.54E-06
IL1RL1	11	3.40	12.10	3.05E-09
TNIP1	7	3.39	76.91	3.51E-10
SDC4	13	3.38	89.15	6.02E-23
CXXC5	7	3.38	62.85	3.99E-42
MARCH10	19	3.37	7.35	6.72E-16
HMGA1	23	3.37	18.53	2.41E-27
RNF19B	2	3.32	22.12	3.41E-23
CLCF1	29	3.32	9.97	5.14E-23
EGFLAM	20	3.32	7.34	4.66E-30
LOC785529	7	3.28	5.97	2.58E-17
TIFA	6	3.27	1.31	1.52E-06
LOC510442	7	3.26	92.94	5.91E-36
TNFAIP6	2	3.26	10.43	6.05E-06
RNF125	24	3.25	4.84	5.27E-09
CMPK2	11	3.18	1.49	1.44E-08
LOC100337034	11	3.16	1.72	4.06E-10
ARRDC2	7	3.15	12.96	2.35E-08
TCF7	7	3.15	12.45	5.42E-08
METRNL	19	3.14	56.28	1.14E-15
LOC100298963	17	3.13	1.68	4.74E-09
BCL2L11	11	3.13	3.44	7.24E-10
LOC100335751	6	3.11	5.01	1.23E-12
TBX3	17	3.08	5.98	2.38E-11
LOC100847896	15	3.07	2.76	0.0322
GPRC5A	5	3.05	1.58	0.000282
BTG2	16	3.02	14.21	0.000522
BHLHE40	22	3.01	296.02	9.00E-13
LOC100138596	12	3.01	13.18	1.25E-14
LOC100139891	8	2.99	6.47	8.39E-16
IRF5	4	2.98	4.81	0.00199
SYNJ2	9	2.98	11.14	3.23E-14
LIMD1	22	2.97	29.21	3.17E-39
IPMK	26	2.97	6.29	1.20E-11
C15H11orf96	15	2.96	45.22	7.93E-06
SLC2A1	3	2.95	24.14	5.24E-10
CD40	13	2.93	16.16	6.04E-13
PPP1R13L	18	2.92	33.98	4.79E-13
LRRC32	15	2.92	39.72	2.32E-18
ZNF385A	5	2.91	13.38	4.62E-06
C23H6orf145	23	2.86	37.59	2.52E-32

JHDM1D	4	2.85	6.47	9.13E-11
ID1	13	2.85	42.05	6.74E-05
VASN	25	2.85	86.32	1.60E-17
EPHA2	2	2.84	42.96	6.43E-24
ZNF469	18	2.84	31.51	4.03E-16
LOC514750	7	2.83	1.92	1.41E-07
TP53I11	15	2.83	2.73	0.0139
KCNK6	18	2.82	12.01	7.40E-05
LIFR	20	2.81	4.02	7.36E-07
LOC100294865	22	2.79	42.74	2.64E-11
FOXA3	18	2.78	1.16	9.67E-06
DLX3	19	2.76	2.38	1.16E-05
GJC1	19	2.72	9.47	2.08E-08
FAM65C	13	2.71	1.58	0.00177
PLK3	3	2.69	36.93	4.21E-18
WNT5A	22	2.68	15.27	0.00143
KLHL21	16	2.68	122.54	3.16E-28
MAFF	5	2.66	10.01	2.54E-11
CSRNP1	22	2.66	16.64	7.24E-05
ADORA2B	19	2.64	1.17	0.000260
SV2C	10	2.63	2.75	7.64E-05
FLT1	12	2.62	34.53	6.21E-10
HMOX1	5	2.59	44.61	0.000302
FHDC1	17	2.59	7.98	4.14E-08
CAMKK1	19	2.57	4.43	9.85E-07
RASL12	10	2.55	11.48	3.44E-12
NR4A2	2	2.55	19.89	5.34E-15
EFNB2	12	2.55	1.85	0.0169
FOXC2	18	2.54	2.68	0.0122
RGS17	9	2.54	7.84	0.000372
LOC100297270	15	2.54	8.49	2.43E-15
TMEM64	14	2.50	12.42	5.43E-21
SAMD4A	10	2.50	36.56	2.51E-23
PHLDB1	15	2.49	106.88	1.92E-20
SGMS2	6	2.48	4.27	1.41E-12
RAB11FIP1	27	2.48	3.89	0.0138
NRN1	23	2.46	141.20	2.20E-07
F2R	10	2.46	4.60	5.77E-05
MAP2K3	19	2.44	97.15	1.10E-29
TSPAN18	15	2.44	2.02	0.0302
HSPA1A	23	2.44	99.80	5.29E-23
FAM20A	19	2.43	14.62	3.20E-07
TICAM1	7	2.43	13.06	1.61E-13
AXIN2	19	2.41	5.84	1.50E-08
ADPRHL1	12	2.41	1.59	0.000936

SPRY4	7	2.40	2.70	6.61E-05
PMEPA1	13	2.40	34.08	8.48E-05
LOC100848842	19	2.40	2.97	4.39E-05
MYLK2	13	2.40	1.56	5.44E-06
STAC2	19	2.39	3.69	1.89E-10
RASSF4	28	2.38	6.39	0.00458
LETM2	27	2.38	3.35	3.17E-06
LOC529061	20	2.38	12.14	6.31E-15
BCL3	18	2.37	24.57	1.95E-05
LOC533821	27	2.37	2.85	0.00271
ITPRIP	26	2.36	33.32	4.08E-23
SPSB1	16	2.36	18.23	3.76E-09
LOC100847176	10	2.36	2.05	3.33E-08
TNFSF9	7	2.36	20.02	3.85E-10
C10H14orf43	10	2.35	28.26	7.42E-10
ETS1	29	2.35	71.85	2.45E-28
HEY2	9	2.35	2.52	0.0327
NFKBID	18	2.34	6.39	3.69E-05
ALDH1A3	21	2.34	49.16	0.000140
CSF1	3	2.34	41.77	4.27E-05
SPATA13	12	2.33	2.52	5.20E-06
SLC20A1	11	2.32	144.24	2.34E-13
SAP25	25	2.31	1.22	0.00936
CCDC97	18	2.31	27.46	2.55E-23
LHFPL2	10	2.31	43.45	6.60E-18
MFSD2A	3	2.30	64.32	1.51E-09
BAIAP2	19	2.29	36.94	2.34E-13
INSIG1	4	2.29	10.83	1.57E-09
TSKU	15	2.29	53.32	4.48E-19
NFKBIE	23	2.28	9.86	9.51E-08
ZSWIM6	20	2.28	16.84	1.98E-17
FURIN	21	2.27	56.03	7.19E-18
IFNAR2	1	2.27	19.25	8.33E-08
CISH	22	2.27	1.25	0.00610
IL18R1	11	2.27	20.15	5.42E-08
FAM102A	11	2.26	6.65	6.33E-06
NDP	X	2.26	1.23	0.00855
MAP3K11	29	2.26	13.18	4.87E-14
PAPD7	20	2.25	44.52	1.03E-19
RASGEF1A	28	2.25	2.41	0.00458
CORO7	25	2.24	90.14	1.77E-12
SKIL	1	2.24	43.21	3.92E-10
CDK17	5	2.23	36.47	1.29E-15
MSX1	6	2.23	2.70	0.000270
EFHD2	16	2.23	91.52	2.09E-13

HRH1	22	2.22	1.32	0.0261
SH3BP2	6	2.22	28.12	7.89E-05
BCOR	X	2.20	47.15	1.24E-19
DNAJB1	7	2.20	37.25	7.69E-21
NAB1	2	2.20	36.48	1.02E-17
AMPD2	3	2.20	49.25	7.57E-15
SERPINE1	25	2.20	3931.72	1.44E-08
PPM1D	19	2.19	16.91	1.52E-17
BST2	7	2.19	2.90	0.0170
HIC1	19	2.17	7.40	2.14E-06
SERTAD1	18	2.17	26.07	1.30E-15
TRIM36	10	2.16	2.55	0.00115
MUC12	25	2.16	3579.28	1.53E-08
EFNB1	X	2.15	27.91	2.98E-10
PDLIM4	7	2.15	42.96	3.93E-09
GFPT2	7	2.14	67.07	9.24E-06
TSHZ3	18	2.14	18.42	1.25E-14
HS3ST2	25	2.12	65.91	0.0500
SELPLG	17	2.11	1.08	0.00133
LOC781048	7	2.11	5.61	1.80E-06
CD3EAP	18	2.11	12.12	9.84E-14
PHLDA1	5	2.09	6.93	6.04E-05
BACH1	1	2.09	34.09	8.48E-07
TWIST2	3	2.08	52.62	1.89E-13
BDKRB2	21	2.07	4.00	0.000382
CEBPD	14	2.07	20.60	0.000248
TGIF1	24	2.06	13.18	1.79E-12
NUMBL	18	2.06	28.06	9.30E-15
ABR	19	2.06	114.21	6.61E-18
SLC35E4	17	2.05	4.86	9.73E-07
KIAA1522	2	2.05	1.73	0.0110
BCAR1	18	2.04	76.38	1.45E-13
TGFB1	18	2.03	71.76	5.10E-16
FSTL3	7	2.02	64.68	1.77E-15
AMD1	9	2.01	125.39	3.96E-08
LFNG	25	2.01	46.64	1.33E-05
DCUN1D2	12	2.01	10.72	5.72E-10
LDLR	7	2.01	158.07	1.05E-11
RASA2	1	2.01	36.98	1.54E-13
SERTAD2	11	2.01	8.86	1.10E-06
STAC	22	2.00	39.17	1.42E-08
RABGEF1	25	2.00	35.97	8.11E-10
MYOZ2	6	-2.00	1.06	3.77E-05
ZNF319	18	-2.01	1.03	0.000731
ZFP1	18	-2.02	1.25	2.12E-05

ZNF280C	X	-2.02	2.25	0.00848
LOC100848732	14	-2.03	1.38	0.000220
RNF34	17	-2.03	6.71	2.32E-05
MID1IP1	X	-2.03	36.02	3.46E-10
LOC100848708	7	-2.03	1.04	0.00302
SHROOM1	7	-2.03	1.21	0.00132
LOC100847387	2	-2.03	2.48	4.02E-06
METTL4	24	-2.03	2.51	0.000310
PRICKLE2	22	-2.04	2.25	0.00485
GADD45A	3	-2.04	80.80	3.83E-14
PAX6	15	-2.04	4.01	0.00256
ATOH8	11	-2.04	13.72	4.18E-07
ZFHX2	10	-2.04	1.51	0.00362
ZNF674	X	-2.05	3.71	2.19E-06
LOC100138767	18	-2.05	5.98	0.0346
RIN1	29	-2.06	2.68	0.000475
PRDM6	7	-2.08	2.06	0.000409
C13H20orf177	13	-2.08	1.45	1.94E-06
CHAMP1	12	-2.08	17.43	1.50E-16
ZNF839	21	-2.08	5.06	8.13E-07
NIM1	20	-2.09	1.18	3.08E-05
RND3	2	-2.10	128.58	8.19E-06
TCEANC	X	-2.10	2.30	2.74E-07
GLI2	2	-2.10	2.57	0.000720
STARD10	15	-2.10	2.21	0.000424
ZNF624	19	-2.10	3.80	6.17E-09
ZNF18	19	-2.11	8.98	6.72E-10
LIPE	18	-2.11	2.28	0.000904
FAM186B	5	-2.11	1.48	9.57E-07
LOC540312	X	-2.13	2.20	0.00129
CACNB2	13	-2.15	2.85	1.49E-07
PRICKLE1	5	-2.16	4.10	0.00188
LOC100849024	22	-2.16	1.50	3.19E-05
SP7	5	-2.16	3.19	0.000575
FGD4	5	-2.17	6.23	4.52E-05
SLC1A3	20	-2.18	1.85	0.0267
NUDT18	8	-2.18	2.18	0.000234
SRL	25	-2.18	4.54	8.39E-06
EMX2	26	-2.18	21.10	1.49E-12
STC2	20	-2.18	5.00	8.35E-09
FOS	10	-2.18	20.33	1.05E-06
MEIS1	11	-2.19	5.56	5.92E-08
LGALS4	18	-2.19	1.96	0.0419
C10H14orf93	10	-2.19	3.03	0.000506
CEP57L1	9	-2.20	3.89	1.38E-06

ZC3H6	11	-2.21	3.19	0.000417
TRIB3	13	-2.22	47.53	1.17E-10
ZNF572	14	-2.23	6.65	1.11E-12
ARHGEF26	1	-2.24	2.17	1.78E-05
NOD1	4	-2.24	1.76	0.000575
SETMAR	22	-2.24	2.48	1.43E-06
SPATA7	10	-2.27	8.69	1.64E-07
LRRC29	18	-2.28	1.14	8.54E-06
LOC100847448	20	-2.28	37.05	7.15E-18
ZNF182	X	-2.28	2.03	5.35E-07
MEOX2	4	-2.30	7.03	7.15E-06
AHR	4	-2.30	4.45	1.58E-10
EID2	18	-2.30	1.04	0.0136
OXSM	27	-2.31	2.32	2.60E-07
PIK3R1	20	-2.32	32.77	2.17E-17
PPP1R3B	27	-2.33	5.25	1.36E-09
ARID5B	28	-2.33	29.80	3.34E-17
LYSMD4	21	-2.34	2.22	1.51E-05
LOC614357	5	-2.36	3.30	0.000969
FAM84B	14	-2.36	8.83	7.19E-05
LOC781565	1	-2.36	16.63	0.0327
NFIL3	8	-2.38	32.27	9.00E-19
ARRDC3	7	-2.38	14.02	1.54E-06
FLRT3	13	-2.38	2.39	9.85E-07
C21H14orf28	21	-2.38	4.81	3.13E-09
ZBTB7C	24	-2.38	1.97	0.00738
LOC513640	28	-2.42	3.15	3.37E-11
CDKN2B	8	-2.42	13.38	0.000234
CYP2U1	6	-2.43	1.42	1.25E-06
CBX2	19	-2.43	1.19	3.95E-06
RAB30	29	-2.44	2.22	9.75E-08
RCOR2	29	-2.44	3.05	0.0129
MAP3K13	1	-2.47	2.14	1.65E-06
MYLK3	18	-2.50	1.33	0.000691
ARRDC4	21	-2.51	7.26	1.88E-07
KCNJ2	19	-2.53	1.94	2.16E-08
CCDC14	1	-2.55	1.81	1.66E-07
MAMSTR	18	-2.59	3.44	8.36E-13
IRX3	18	-2.62	5.51	7.97E-12
JUB	10	-2.63	5.68	1.15E-08
C8H9orf150	8	-2.64	7.09	3.60E-11
RWDD3	3	-2.66	3.00	7.40E-10
TRIM66	15	-2.69	1.05	1.99E-06
ZNF789	25	-2.70	1.13	1.46E-09
PPM1K	6	-2.74	53.07	4.27E-07

NUAK1	5	-2.76	49.94	1.11E-09
SESN2	2	-2.83	32.72	7.26E-26
IRX5	18	-2.86	8.56	3.08E-18
CBX4	19	-2.89	12.00	7.93E-18
NR1D1	19	-2.93	7.96	2.24E-16
FAM46B	2	-3.06	6.20	5.09E-09
LOC100847770	13	-3.06	1.42	1.35E-06
C3H1orf51	3	-3.07	1.48	8.62E-09
ZNF22	28	-3.09	3.92	4.05E-20
KIAA0226L	12	-3.12	3.65	0.00126
SPRY1	17	-3.22	3.25	2.97E-11
PTPDC1	8	-3.41	2.43	2.08E-17
THNSL2	11	-3.47	1.06	0.00876
LRIG3	5	-3.60	28.54	7.52E-14
PDK4	4	-4.59	1.10	0.000178
CHAC1	10	-4.79	14.59	9.12E-43
RNF122	27	-5.37	2.33	0.0357
TXNIP	3	-5.43	45.34	1.30E-15
LOC100848155	3	-5.62	5.72	3.01E-11
KCNE4	2	-11.08	3.52	4.57E-10
