# Supplementary material for: Differential responsiveness of Holstein and Angus dermal fibroblasts to LPS challenge occurs without major differences in the methylome
Source: BMC Genomics. 2016 Mar 24;17:258. doi: 10.1186/s12864-016-2565-x (PMC4806443; doi:10.1186/s12864-016-2565-x)
Supplement: Additional file 2: — Genes displaying differential gene expression (FDR < 0.05; CPM > 1; 2 ≤ FC ≤ -2) due to LPS at hour 8 as compared to hour 0. A positive fold change indicates higher expression at hour 8 compared to hour 0. CPM = counts per million. FDR = false discovery rate. Data shown for comparisons with an FDR < 0.05; CPM > 1; FC ≥ 2. (PDF 63 kb) [file 12864_2016_2565_MOESM2_ESM.pdf]

Gene	Chromosome	FC	CPM	FDR
CCL20	2	3147.31	2.53	1.12E-18
SAA3	29	1145.59	3.87	1.40E-23
IL8	6	387.80	26.47	6.01E-43
ISG15	16	308.32	6.23	1.72E-29
MX2	1	156.60	1.53	1.85E-15
CCL5	19	132.81	25.79	2.62E-22
OAS1	17	128.69	14.30	3.52E-17
IL6	4	124.12	31.73	1.69E-36
CFB	23	118.45	48.71	7.77E-21
OAS2	17	65.58	1.86	4.19E-12
VCAM1	3	44.98	8.08	1.69E-15
CXCL6	6	44.95	257.47	2.19E-26
RSAD2	11	44.30	1.13	7.32E-18
ZBP1	13	32.06	1.41	2.78E-16
CXCL2	6	31.62	38.77	1.92E-65
PTX3	1	29.95	5.78	7.42E-12
RND1	5	23.85	11.10	6.58E-09
IFIH1	2	23.75	5.94	4.28E-21
BST2	7	21.10	21.08	3.07E-18
STRA6	21	20.32	3.65	4.34E-09
IFI27	21	17.91	2.01	3.72E-05
CCL2	19	14.57	82.85	2.73E-14
TNFAIP3	9	13.82	5.56	2.62E-22
OCLN	20	13.77	1.43	1.43E-24
LOC508347	3	11.58	1.55	6.92E-12
CYP3A4	25	10.56	49.32	6.39E-11
HAS2	14	10.47	4.31	7.02E-21
SLCO4A1	13	10.28	1.15	2.58E-09
CDA	2	10.23	1.37	1.89E-08
TGM3	13	9.44	21.58	2.30E-06
NFKBIA	21	9.34	31.14	1.37E-33
MX1	1	8.86	19.67	1.17E-08
PDPN	16	8.68	6.31	2.97E-13
PTGS2	16	8.48	39.13	3.13E-19
LOC100138376	16	8.48	24.92	2.40E-18
LOC100848019	9	8.15	36.82	6.15E-13
RTP4	1	7.89	1.55	2.55E-12
OLR1	5	7.81	74.82	1.84E-07
ARNTL2	5	7.80	1.73	6.66E-09
NCALD	14	7.52	1.65	7.79E-09
LOC511531	3	7.42	6.85	9.30E-11
DHX58	19	6.77	3.56	4.69E-08
SERPINB2	24	6.53	10.71	3.34E-07

GBP5	3	6.53	5.67	2.69E-15
SOD2	9	6.46	10.25	6.38E-09
MXRA5	X	6.36	6.44	2.96E-05
LOC785403	8	6.11	1.83	8.27E-06
DRD3	1	5.85	1.81	2.69E-15
SDPR	2	5.76	1.15	6.49E-07
IL1A	11	5.71	1.06	2.00E-07
PION	4	5.70	4.10	1.30E-14
PSMB9	23	5.45	1.53	1.76E-13
CMPK2	11	5.36	2.39	5.91E-08
LOC507055	3	4.91	2.96	8.37E-15
TNIP1	7	4.80	107.03	9.66E-13
TLR2	17	4.75	2.92	1.92E-07
LOC782264	12	4.25	2.69	6.87E-07
PDE4B	3	4.22	9.87	8.53E-18
IRF1	7	4.20	10.31	1.25E-18
MT2A	18	4.19	25.35	0.000323
IL1RL1	11	4.18	15.03	2.33E-12
CYP7B1	14	4.18	5.27	2.19E-09
BIRC3	15	4.15	30.05	4.96E-16
LOC100848038	7	4.14	22.49	1.76E-21
CD40	13	4.09	22.08	9.76E-18
MARCKSL1	2	4.01	9.00	2.85E-07
PLA2G4A	16	3.97	42.52	2.85E-20
ADORA2B	19	3.96	1.68	7.65E-07
TMEM100	19	3.93	1.20	2.20E-05
ALDH1A3	21	3.67	72.38	3.81E-07
CBLN3	10	3.62	2.72	1.98E-11
C2	23	3.57	1.89	0.00113
SLC2A1	3	3.56	29.36	7.85E-20
CDO1	10	3.55	4.28	2.19E-06
CDC42EP2	29	3.49	1.21	2.00E-06
RELB	18	3.47	16.08	2.53E-11
ESM1	20	3.46	46.30	0.0485
NR4A3	8	3.37	9.18	4.07E-09
F2R	10	3.36	6.13	1.47E-07
PAQR7	2	3.36	1.49	0.0259
NFKBIZ	1	3.23	18.36	3.60E-13
BDKRB1	21	3.20	4.84	0.00152
GFPT2	7	3.14	93.27	9.29E-08
CYP3A5	25	3.11	83.33	4.85E-08
CTSW	29	3.03	1.43	0.0181
BDKRB2	21	2.93	5.40	2.25E-08
LOC512486	3	2.91	6.20	1.47E-07
GPR77	18	2.89	1.24	1.27E-06

ATP8A2	12	2.88	6.31	1.51E-07
WNT5A	22	2.88	16.95	8.35E-06
EPSTI1	12	2.86	8.51	5.89E-05
TAP1	23	2.85	5.73	4.51E-06
NFKB2	26	2.80	67.61	1.73E-13
SERPINE2	2	2.76	155.74	1.86E-07
FAM65C	13	2.75	1.69	0.0188
SLFN11	19	2.74	10.48	4.74E-09
AVIL	5	2.72	3.18	2.14E-05
NR4A2	2	2.72	21.97	1.76E-13
CTSC	29	2.72	61.04	6.99E-17
FILIP1	9	2.63	6.30	3.12E-06
ZC3H12A	3	2.62	10.36	2.10E-07
CILP2	7	2.62	1.95	2.16E-06
ERAP2	7	2.62	10.03	7.35E-07
PLAUR	18	2.60	114.65	2.59E-14
MIR147	10	2.58	1.37	0.000422
PDXK	1	2.57	21.08	3.28E-08
GPRC5A	5	2.57	1.48	0.00308
XDH	11	2.57	270.51	1.39E-10
LOC100847310	18	2.55	122.93	1.77E-13
PCDH11Y	X	2.55	40.90	0.0193
PSMB8	23	2.53	5.23	1.02E-07
IL15RA	13	2.53	17.05	4.13E-15
AXIN2	19	2.52	6.36	2.07E-05
TGFBI	7	2.52	16.49	0.00795
TMEM158	22	2.50	14.67	0.0109
BNC2	8	2.48	7.78	0.0292
CPM	5	2.46	18.20	0.0255
C10H15orf48	10	2.46	7.13	0.000559
CA2	14	2.43	54.79	1.85E-09
MYO5C	10	2.42	4.11	0.00294
RASSF4	28	2.41	6.80	0.0332
SIPA1L2	28	2.40	10.42	1.02E-07
ADPRHL1	12	2.40	1.69	0.00252
SV2C	10	2.40	2.72	4.38E-05
LOC100196897	14	2.38	4.83	0.000254
ITGA2	20	2.38	2.12	0.0110
IER3	23	2.36	40.46	1.26E-11
CASP4	15	2.36	36.42	1.35E-10
IFI16	3	2.35	19.78	5.82E-18
PDE3A	5	2.31	20.27	3.40E-16
VAMP1	5	2.31	3.40	3.58E-05
LOC100849043	20	2.31	2.41	0.00537
PARP9	1	2.30	10.48	7.85E-06

SPATA13	12	2.30	2.64	0.00322
RGS16	16	2.28	3.91	0.00373
GPC6	12	2.28	16.95	7.81E-05
NFKB1	6	2.27	23.53	3.71E-10
CCDC19	3	2.27	1.73	0.0131
RGS17	9	2.27	7.65	0.000447
YOD1	16	2.24	2.66	3.11E-05
SPP1	6	2.24	37.30	0.0112
LOC533821	27	2.24	2.90	0.00669
ARNTL	15	2.23	25.08	8.88E-21
VEGFC	27	2.22	49.77	6.24E-11
DDX58	8	2.21	10.92	0.00132
CEBPD	14	2.21	22.75	0.000196
PID1	2	2.21	26.75	2.45E-08
RNF125	24	2.21	3.86	0.000340
IFNAR2	1	2.17	19.70	2.86E-07
MAFF	5	2.17	9.15	4.06E-06
ANGPTL4	7	2.16	28.45	0.00153
NR4A1	5	2.15	23.67	0.00106
IFIT3	26	2.15	3.83	0.00680
CLMP	15	2.14	45.96	8.33E-13
RHOJ	10	2.13	12.59	0.0158
LOC100848842	19	2.13	2.89	0.00110
ICAM1	7	2.13	6.07	0.0484
SYNJ2	9	2.12	9.23	3.86E-06
RNF213	19	2.11	75.71	0.00194
CHST11	5	2.09	5.25	1.25E-06
PHLDA1	5	2.07	7.27	7.09E-06
MAMDC2	8	2.07	3.53	0.00122
ECE1	2	2.06	44.17	0.000755
LOC540363	17	2.06	1.30	0.0134
FGF7	10	2.04	57.54	6.52E-08
SOCS2	5	2.04	4.82	0.00581
RSPO3	9	2.02	16.03	0.000294
GLDC	8	2.02	19.84	1.67E-09
LOC100335751	6	2.02	3.88	4.78E-05
HIF1A	10	2.01	139.99	1.69E-06
IL18R1	11	2.01	19.56	7.79E-09
PSD2	7	-2.00	2.22	0.00591
RPS6KA1	2	-2.01	2.29	0.0417
FNDC4	11	-2.02	5.26	0.0145
TOX	14	-2.02	1.85	0.00631
LIMS2	2	-2.03	10.38	0.0360
FBXO10	8	-2.04	1.16	0.000417
SPNS2	19	-2.06	3.66	0.00213

MAPK11	5	-2.06	4.47	3.06E-05
ADAM33	13	-2.07	238.50	1.27E-18
JUP	19	-2.07	34.24	3.33E-06
CBFA2T3	18	-2.07	11.83	0.00344
LZTS1	8	-2.07	4.84	3.17E-06
KLHL13	X	-2.07	4.97	0.00103
NHS	X	-2.08	5.31	0.000161
TRAF5	16	-2.08	12.58	2.69E-05
FOS	10	-2.08	21.75	3.97E-05
RAB3IL1	29	-2.10	5.53	8.74E-06
FAM13C	28	-2.13	19.35	0.0299
SARDH	11	-2.14	4.86	4.37E-06
DACT1	10	-2.15	13.86	0.00993
FGD4	5	-2.15	6.58	7.58E-07
LAMB3	16	-2.18	3.32	0.0101
AQP11	29	-2.18	1.78	0.0394
PPL	25	-2.18	16.39	0.0181
ZBTB7C	24	-2.20	2.12	0.0236
SHANK1	18	-2.21	4.82	0.000460
LOC540312	X	-2.22	2.28	0.000196
MGC148692	6	-2.23	1.59	0.0257
C3H1orf183	3	-2.24	2.27	0.000232
LAMA5	13	-2.30	20.39	3.84E-05
STARD10	15	-2.31	2.26	7.22E-06
TYRO3	10	-2.33	27.95	2.27E-20
CHD5	16	-2.36	2.66	0.000115
GFRA4	13	-2.38	9.13	6.45E-11
LOC100848739	14	-2.39	5.15	0.000352
PDK4	4	-2.43	1.34	0.0397
KIF5A	5	-2.46	2.19	1.81E-06
LOC100847582	19	-2.49	19374.58	0.00234
COL1A1	19	-2.56	14563.18	0.00215
AMIGO2	5	-2.58	36.80	5.77E-12
FGFR3	6	-2.60	2.81	0.00210
SP7	5	-2.62	3.17	0.00104
CDH6	20	-2.62	1.03	2.82E-07
AHNAK2	21	-2.62	24.84	8.91E-10
C19H17orf109	19	-2.65	1.01	1.09E-06
DBP	18	-2.77	3.44	8.14E-09
SEMA5B	1	-2.84	35.83	0.00711
LMCD1	22	-3.02	36.02	1.07E-15
SESN3	15	-3.02	10.44	1.89E-12
PAK1	29	-3.05	37.77	9.97E-26
TCEA3	2	-3.14	1.44	3.91E-07
PTCHD1	X	-3.32	1.15	1.77E-06

RSPO2	14	-3.56	1.12	0.000337
EGR1	7	-3.66	20.44	2.18E-07
HDAC9	4	-3.68	10.22	3.61E-07
PAK6	10	-4.62	1.07	2.15E-05
