# Supplementary material for: Differential responsiveness of Holstein and Angus dermal fibroblasts to LPS challenge occurs without major differences in the methylome
Source: BMC Genomics. 2016 Mar 24;17:258. doi: 10.1186/s12864-016-2565-x (PMC4806443; doi:10.1186/s12864-016-2565-x)
Supplement: Additional file 3: — Differentially expressed genes (FDR < 0.05; CPM > 1; 2 ≤ FC ≤ -2) between Holstein and Angus fibroblast cultures exposed to 100ng/ml LPS for 0, 2, and 8 h. A positive fold change indicates higher expression in Holstein cultures. CPM = counts per million. FDR = false discovery rate. (PDF 207 kb) [file 12864_2016_2565_MOESM3_ESM.pdf]

Hour 0				
**Gene**	**Chromosome**	**FC**	**CPM**	**FDR**
KRT17	19	1362.72	1.07	4.21E-05
KRT5	5	62.23	1.27	0.000170
LOC100296277	X	18.65	3.54	0.00554
PAX2	26	18.60	1.23	1.33E-07
ISLR2	21	13.12	1.62	1.26E-11
MATN3	11	11.89	15.57	0.00391
AFAP1L2	26	11.52	3.85	6.12E-09
FAM131B	4	11.49	2.07	4.46E-16
MAPK13	23	11.19	1.60	3.60E-08
CD200	1	10.27	2.38	1.66E-05
RPS27	17	9.41	22.41	0.000346
HEYL	3	9.01	6.53	0.00390
LOC615559	21	8.72	1.83	6.41E-05
HES4	16	7.44	1.42	4.63E-08
FAM150A	14	7.32	1.54	0.00613
DSC2	24	7.04	19.39	0.00171
TNFSF18	16	6.51	56.25	4.46E-16
GDAP1L1	13	6.38	1.87	0.00424
GALNTL2	1	6.13	4.66	3.96E-10
USP43	19	5.86	5.05	1.35E-07
ACAN	21	5.84	48.45	0.000281
CLIC5	23	5.40	1.14	9.94E-06
CFH	16	5.38	15.59	0.000299
SLC6A6	22	5.13	11.83	3.58E-07
RBPMS2	10	5.12	1.31	0.00514
MEST	4	4.80	4.51	0.0126
TSPAN2	3	4.68	1.43	7.31E-05
CARD11	25	4.62	2.53	0.0346
LOC781004	16	4.55	1.35	0.00105
GIPC3	7	4.45	7.23	6.60E-09
DSC3	24	4.44	52.30	0.00598
TNFSF4	16	4.40	1.19	3.40E-07
PPP1R14A	18	4.16	1.34	3.30E-05
GLRB	17	4.11	2.69	0.00326
GAS6	12	4.07	7.24	8.90E-11
TPPP	20	4.06	2.01	2.76E-07
IGFBP7	6	3.93	25.80	0.000386
GREB1	11	3.93	1.82	0.0200
LOC506672	2	3.91	3.84	0.0118
WDR35	11	3.87	75.24	2.60E-14
GPX3	7	3.85	14.64	0.000142
PIK3IP1	17	3.80	3.97	0.00580
LOC100300760	16	3.80	1.07	0.0212

CNKSR1	2	3.77	1.01	0.00484
RASGRP2	29	3.75	5.48	0.000140
ELMO3	18	3.75	1.75	0.0285
PNPLA1	23	3.75	1.48	0.000281
RASSF5	16	3.71	3.71	0.000678
IGF2BP2	1	3.69	4.29	0.000128
PEAR1	3	3.66	6.25	3.67E-08
ANKH	20	3.66	148.03	1.77E-11
GPR133	17	3.64	3.98	1.60E-06
COL8A1	1	3.63	10.18	0.00689
LOC100847471	8	3.60	1.48	4.86E-06
MYCL1	3	3.59	1.09	1.82E-07
COL11A1	3	3.57	846.50	2.89E-05
LOC100847123	26	3.53	1.02	0.00152
LOC535166	14	3.53	4.46	0.00451
DMPK	18	3.50	11.10	8.94E-13
CABLES1	24	3.45	8.34	2.50E-13
SEMA5B	1	3.44	51.55	2.31E-14
MGP	5	3.39	85.23	1.33E-05
BCAS1	13	3.38	4.03	0.0387
JAKMIP3	26	3.37	3.09	7.80E-05
SEMA7A	21	3.33	2.96	0.0152
OLFM2	7	3.29	7.09	0.000134
SLC6A16	18	3.19	3.37	7.37E-05
CASQ2	3	3.15	2.11	0.00186
STX1A	25	3.13	2.92	5.32E-06
C18H19orf33	18	3.13	1.62	9.56E-05
SEMA5A	20	3.12	3.55	0.000215
AGRN	16	3.11	1.06	0.000328
LOC100848128	11	3.09	2.93	0.00725
TNFRSF19	12	3.08	1.01	0.00116
FAIM2	5	3.07	3.68	0.0459
LOXL3	11	3.05	22.56	3.84E-08
SUSD4	16	3.03	1.37	0.00420
KRBA1	4	3.02	3.12	0.000128
PAK6	10	3.00	1.71	0.0391
SPINT2	18	3.00	18.81	7.67E-14
NPDC1	11	3.00	6.40	0.0164
ABCA1	8	2.98	6.52	0.00443
ODZ4	29	2.98	6.49	0.00190
TLR4	8	2.98	2.48	0.0122
LOC100336868	16	2.97	2.03	0.00472
FAM83H	14	2.93	8.30	0.000931
SPEG	2	2.91	18.33	2.02E-09
ENHO	8	2.90	1.09	0.0130

LOC100848155	3	2.89	9.95	9.96E-09
RDH10	14	2.85	45.10	2.87E-09
PROS1	1	2.83	107.08	0.0103
PM20D1	16	2.82	7.72	0.000127
PMEPA1	13	2.81	20.54	3.56E-09
MGC148692	6	2.81	2.13	8.55E-05
LOC539821	16	2.81	1.51	9.56E-05
LOC100847602	5	2.80	9.57	2.43E-07
BNC2	8	2.78	4.34	0.000224
FAM105A	20	2.75	2.26	0.00341
CCL26	25	2.73	2.44	0.0107
LOC100848243	12	2.72	1.21	0.0144
ATP6AP1L	7	2.72	4.31	0.000403
FAM43A	1	2.69	3.10	0.00477
FBXO32	14	2.69	3.06	0.00329
SLC29A2	29	2.69	6.90	0.000841
LOC789525	18	2.68	1.02	0.0160
RAB11FIP1	27	2.67	2.29	0.00359
LOC100848899	3	2.66	1.99	0.0234
OLR1	5	2.66	16.51	5.78E-06
B4GALNT1	5	2.66	1.81	0.000145
ITGA11	10	2.65	222.49	5.86E-05
C8H9orf103	8	2.65	2.40	0.000105
MOCOS	24	2.64	3.42	0.0451
PRSS48	17	2.62	1.30	0.0248
LOC100336584	24	2.61	1.33	0.00492
FGF10	20	2.59	17.23	0.0226
CDH17	14	2.59	1.45	0.00746
SEPT4	19	2.57	3.23	0.00170
MRAP2	9	2.56	1.26	0.0146
SPTBN4	18	2.55	1.14	0.00174
FNDC4	11	2.54	6.84	3.67E-08
LOC100847694	X	2.54	1.87	0.00210
NKX2-2	13	2.53	1.94	0.00237
LOC100847829	X	2.53	1.47	0.00375
LOC789485	7	2.53	104.67	0.00308
LOC100296463	25	2.53	11.09	6.68E-07
LOC777601	X	2.52	1.47	0.00382
SDC1	11	2.51	68.30	3.99E-08
HSPB6	18	2.50	108.69	2.58E-07
LOC100847766	X	2.50	1.48	0.00435
LOC100848300	18	2.50	1.44	0.0390
SLC25A13	4	2.50	5.00	0.0340
LMTK3	18	2.49	2.58	7.55E-05
TGFB2	16	2.49	47.54	4.10E-06

RGS5	3	2.49	1.77	0.0115
LOC100299061	X	2.48	1.59	0.00321
ACER2	8	2.47	1.34	0.00339
RGS4	3	2.46	12.56	0.000536
KCTD12	12	2.46	13.20	0.0129
TRPV4	17	2.45	4.46	0.00270
TXNIP	3	2.45	78.51	1.87E-11
LOC615989	3	2.45	1.51	0.0129
ALDOC	19	2.44	16.47	0.00477
TRAF1	8	2.44	2.05	0.000247
COLQ	1	2.44	1.00	0.0376
ZMYND15	19	2.44	4.71	0.0163
CYP3A4	25	2.43	8.29	0.00155
SPON1	15	2.43	28.08	7.92E-06
TMEM108	1	2.42	1.11	0.00486
NOXA1	11	2.41	3.34	0.00443
IRF5	4	2.41	2.48	0.0346
CHCHD6	22	2.41	1.37	0.00120
RFX2	7	2.39	3.67	0.000172
RASGRF2	7	2.39	4.38	0.000955
LOC100848206	X	2.39	4.44	0.00209
CHRD	1	2.37	6.95	0.00280
PRKCG	18	2.37	1.03	0.00227
CHPF	2	2.37	126.62	4.78E-08
PRUNE2	8	2.36	15.10	0.000316
TCP11L2	5	2.36	6.46	0.0110
IGFBP3	4	2.36	101.56	0.00375
SLC13A4	4	2.35	3.02	0.00370
OSCAR	18	2.34	3.83	0.0346
ITGBL1	12	2.34	20.72	9.81E-06
LOC100848191	18	2.34	104.90	6.12E-08
LOC100337435	21	2.34	4.73	0.0133
LOC526745	13	2.34	4.19	0.0493
ABCA6	19	2.33	5.77	0.0365
CNNM2	26	2.33	27.68	0.000142
LOC509006	7	2.32	58.63	3.78E-12
FN1	2	2.31	11274.95	0.00270
C24H18orf1	24	2.31	13.72	0.000230
CATSPERG	18	2.31	14.70	3.60E-08
COL4A2	12	2.29	483.76	2.47E-08
OBSL1	2	2.28	16.35	0.00144
NAT14	18	2.28	2.61	0.00204
LOC788414	2	2.27	1.82	0.0250
RHBDL2	3	2.27	2.94	0.00215
CES2	18	2.27	3.27	0.0202

MRVI1	15	2.26	23.22	8.57E-06
UNC5B	28	2.26	6.45	1.28E-05
EFEMP1	11	2.26	83.31	0.00624
VWCE	29	2.25	1.04	0.0115
SDR42E1	18	2.25	1.10	0.00658
LOC100848636	5	2.25	5.34	0.000700
CACNA1A	7	2.25	14.15	0.0134
SYN2	22	2.24	1.99	0.0403
SLC16A11	19	2.24	9.85	0.00413
MICALL2	25	2.24	5.56	3.69E-06
KLHL24	1	2.22	5.60	0.00375
CDKN2B	8	2.21	19.42	7.90E-05
TMEM26	28	2.20	10.74	0.000156
SARM1	19	2.20	4.50	0.0312
CASK	X	2.20	122.09	0.000593
ACTC1	10	2.20	1.14	0.0155
LOC100848739	14	2.18	7.07	0.000579
TNFRSF25	16	2.17	2.68	0.000298
FAM198B	17	2.17	6.83	0.000773
HAVCR2	7	2.17	1.46	0.0266
FAM115C	4	2.16	4.45	0.00243
P4HA3	15	2.16	9.94	0.00220
PKLR	3	2.16	1.28	0.0165
AQP11	29	2.16	2.37	0.00954
RAB20	12	2.16	3.22	0.00460
EGLN3	21	2.15	9.62	0.0199
C13H20orf112	13	2.15	2.30	0.0352
PALMD	3	2.15	1.06	0.0455
CELSR3	22	2.15	2.09	0.0191
DTNA	24	2.14	4.06	0.00461
C12H13orf15	12	2.14	47.59	9.42E-06
ARHGAP29	3	2.13	96.02	6.87E-09
ABCA4	3	2.13	1.37	0.00233
HSF4	18	2.13	8.05	0.0325
SCG5	10	2.13	9.61	1.64E-05
KIAA1462	13	2.12	37.67	2.74E-08
FADS6	19	2.12	3.87	0.0197
APOE	18	2.12	10.27	0.00504
IL18	15	2.12	19.31	4.38E-06
HCRTR1	2	2.11	1.56	0.0379
ENC1	20	2.11	119.36	3.94E-06
LOC100848886	21	2.11	1.00	0.0455
MCF2L	12	2.11	1.08	0.0287
PRR5L	15	2.11	13.19	0.00304
MMP15	18	2.10	2.14	0.00565

JAG1	13	2.10	215.31	0.000209
LOC100141258	25	2.10	1.84	0.0398
COL4A1	12	2.10	1680.55	7.31E-05
WISP1	14	2.09	22.91	1.59E-05
GLULP	2	2.09	8.25	2.38E-05
ARHGEF5	4	2.09	7.61	0.00831
SST	1	2.09	60.18	0.00414
KCNK6	18	2.09	6.44	0.000507
TGFB3	10	2.08	31.39	1.32E-08
KIAA1522	2	2.08	1.16	0.0299
ACY1	22	2.08	8.48	0.000359
C26H10orf92	26	2.07	1.88	0.00490
YPEL2	19	2.07	12.03	0.000116
TCF7	7	2.06	6.15	0.00460
MICAL2	15	2.06	192.10	2.05E-08
NDRG2	10	2.05	4.52	0.0334
GRAMD1B	15	2.05	3.51	0.00581
C29H11orf80	29	2.05	12.21	0.000309
GPR63	9	2.04	11.45	0.00265
CPM	5	2.04	10.24	0.0298
FAP	2	2.04	65.54	1.92E-05
CBFA2T3	18	2.03	15.53	0.000435
SOX4	23	2.03	2.04	0.0419
LOC100849059	7	2.03	2.72	0.00735
AGMO	4	2.03	30.72	7.44E-11
LOC615589	5	2.03	1.18	0.0312
CST3	13	2.02	101.56	0.00107
CAPRIN2	5	2.02	10.91	0.00701
PPM1N	18	2.02	1.65	0.00993
GDAP1	14	2.02	3.90	0.0108
NMRAL1	25	2.02	10.19	0.00128
HSD11B1L	7	2.02	3.66	0.00216
CTSZ	13	2.02	67.98	6.12E-07
LOC100336905	X	2.02	4.99	0.00598
PAG1	14	2.02	3.32	0.00234
SIRT3	11	2.01	2.49	0.00492
VSIG8	3	2.01	3.01	0.000992
LOC786974	20	2.01	9.14	8.16E-06
ELOVL7	20	2.01	25.01	0.0285
FAM84B	14	2.01	12.71	0.00954
FLT1	12	2.00	19.58	0.000128
METRN	25	2.00	2.05	0.0222
CDC6	19	-2.00	11.86	0.00427
PLXNA2	16	-2.00	9.17	0.00459
GCLM	3	-2.00	24.00	1.62E-07

DNA2	28	-2.01	8.32	0.00839
NFATC2	13	-2.01	1.10	0.0446
FAM13C	28	-2.01	25.61	0.000832
KIAA1524	1	-2.01	20.71	0.000261
NEIL3	27	-2.02	8.22	0.00578
DNER	2	-2.02	1.93	0.0328
CDKN2D	7	-2.02	7.60	0.00877
CENPK	20	-2.03	19.00	0.000644
BARD1	2	-2.04	7.13	0.00337
MCM5	5	-2.04	71.71	0.00103
GLIS1	3	-2.04	6.90	0.0465
SPC24	7	-2.04	36.77	0.000268
CYP26B1	11	-2.04	26.78	0.00157
FANCI	21	-2.04	40.73	0.00115
CCDC34	15	-2.05	9.24	0.000624
MCM3	23	-2.05	72.23	0.00110
CDCA5	29	-2.05	13.23	0.00495
LOC618297	4	-2.06	33.79	0.000194
LIPG	24	-2.06	59.21	5.75E-12
CCDC18	3	-2.06	6.64	0.00208
FANCD2	22	-2.07	53.89	0.00110
PMF1	3	-2.07	10.61	0.000769
AQPEP	10	-2.08	1.44	0.00884
NPR3	20	-2.08	62.74	0.000224
MPPED2	15	-2.08	1.09	0.0209
HMGB2	8	-2.09	37.75	7.31E-05
EPHB4	25	-2.09	47.89	6.33E-06
INCENP	29	-2.09	50.12	0.000278
LOC782598	21	-2.09	12.79	0.000380
PCOLCE2	1	-2.09	7.34	0.00374
C22H3orf23	22	-2.10	1.15	0.0169
LOC100848011	22	-2.10	4.06	0.000892
TPX2	13	-2.10	99.36	3.81E-05
GTSE1	5	-2.10	39.11	0.000372
PHF19	8	-2.11	30.57	0.000731
LOC100848095	5	-2.11	12.95	0.00169
FAM110A	13	-2.11	3.76	0.00485
HSPB8	17	-2.11	71.90	1.18E-10
LOC100847559	5	-2.11	8.99	0.00482
LOC100301478	X	-2.12	13.08	7.31E-05
STIL	3	-2.12	13.95	0.000705
RELL1	6	-2.12	19.05	5.29E-08
TMPO	5	-2.13	76.94	8.59E-05
IGFBP4	19	-2.14	457.73	0.00485
GDA	8	-2.14	1.99	0.0323

ACSS3	5	-2.15	14.31	3.68E-05
FIGNL1	4	-2.15	6.72	0.00835
ADRB2	7	-2.15	9.63	0.000424
SGOL1	1	-2.15	12.33	0.000548
NCAPD2	5	-2.16	78.07	0.000112
TSPAN13	4	-2.16	45.17	3.13E-13
CLSPN	3	-2.16	22.79	0.00108
DIAPH3	12	-2.16	86.50	0.000227
GAS1	8	-2.17	3.69	0.00459
MELK	8	-2.17	30.23	0.000487
SLC24A5	10	-2.17	11.61	0.00220
SMC2	8	-2.18	91.02	6.96E-05
MET	4	-2.18	18.62	1.12E-07
CENPQ	23	-2.19	19.66	0.000678
LOC100847497	X	-2.19	1.66	0.00359
TAP1	23	-2.20	2.92	0.0235
MASP1	1	-2.20	35.05	0.0149
AURKB	19	-2.20	28.92	9.22E-05
FAM54A	9	-2.20	21.21	4.07E-05
LNX1	6	-2.21	3.42	0.000713
PBK	8	-2.21	65.77	4.93E-05
NCAPG	6	-2.21	80.56	8.68E-05
MYBL2	13	-2.21	92.11	0.000142
SPC25	2	-2.22	11.90	0.000421
CENPT	18	-2.22	26.46	0.00101
NCAPG2	4	-2.22	71.34	0.000297
ESRRG	16	-2.22	4.47	0.00326
EXO1	16	-2.22	9.16	0.00114
RNF144B	23	-2.23	2.21	0.00204
SDK2	19	-2.23	9.40	0.00738
KIF18A	15	-2.23	8.70	0.000177
C10H15orf23	10	-2.24	18.83	5.05E-05
MIS18BP1	21	-2.25	34.99	0.000403
WHSC1	6	-2.25	56.83	8.78E-05
IQGAP3	3	-2.25	78.53	0.000167
CADM1	15	-2.25	5.19	0.000210
GPR176	10	-2.27	1.48	0.0197
CACNA1C	5	-2.27	3.90	0.000548
TTK	9	-2.27	5.39	0.00132
KIF4A	X	-2.27	40.36	6.41E-05
DEPDC1B	20	-2.28	6.58	0.00109
KNTC1	17	-2.28	50.77	0.000119
PIR	X	-2.28	7.37	0.000171
SCD	26	-2.28	22.62	2.58E-08
CXHXorf30	X	-2.29	1.14	0.0246

EZH2	4	-2.29	51.03	8.55E-05
CCBE1	24	-2.29	9.04	0.000378
FBXO5	9	-2.29	10.96	0.00103
MND1	17	-2.31	8.79	0.000915
SKA1	24	-2.31	13.26	0.000311
C5H12orf48	5	-2.32	11.79	4.61E-05
KPNA2	19	-2.32	197.73	2.58E-05
UHRF1	7	-2.33	54.33	0.000287
MAN1C1	2	-2.33	5.45	0.0169
LOC100138767	18	-2.33	8.24	0.0265
PION	4	-2.34	1.21	0.00190
SCARA3	8	-2.34	6.22	0.00175
CHST1	15	-2.34	9.06	4.99E-05
FGFR3	6	-2.35	3.94	0.0128
CKAP2	12	-2.35	131.72	0.000268
CDCA8	3	-2.35	27.09	9.73E-05
CEP72	20	-2.36	12.23	0.00176
KIF26B	16	-2.36	38.43	0.00132
LMNB2	7	-2.37	20.27	0.000195
CDCA4	21	-2.37	5.26	0.00363
ERCC6L	X	-2.38	9.00	0.000206
LOC100847721	9	-2.38	20.35	0.000140
LOC615206	26	-2.39	2.41	0.00213
LOC100849043	20	-2.39	1.44	0.00132
LOC100336690	5	-2.39	20.18	0.000100
KIF22	25	-2.39	45.84	3.83E-05
NUF2	3	-2.40	38.29	4.46E-05
MAD2L1	6	-2.40	31.83	1.70E-05
BIRC5	19	-2.40	39.75	9.92E-05
HMMR	7	-2.40	53.16	1.64E-05
FAM72A	16	-2.40	7.89	0.000360
SKA3	12	-2.41	23.00	2.29E-05
TGFBR3	3	-2.41	32.41	4.73E-06
RRM2	11	-2.41	233.80	0.000234
CCDC99	20	-2.42	26.18	0.000204
APCDD1	24	-2.42	1.25	0.00546
SULT1B1	6	-2.42	1.44	0.00174
CKAP2L	11	-2.42	27.62	6.07E-05
ODZ2	7	-2.43	2.52	0.00128
FABP3	2	-2.43	9.12	0.000100
SMC4	1	-2.45	116.08	1.07E-05
OIP5	10	-2.45	11.57	0.000465
ANLN	4	-2.45	305.59	3.79E-06
LOC618307	3	-2.45	1.19	0.00984
ASF1B	7	-2.46	15.91	0.000129

RAD51AP1	5	-2.47	13.53	0.000385
ESCO2	8	-2.47	18.72	7.37E-05
HMOX1	5	-2.48	25.47	2.57E-10
CEP55	26	-2.48	27.75	5.46E-05
STMN1	2	-2.48	64.21	1.66E-05
LOC786089	5	-2.48	2.12	0.00549
KIFC1	23	-2.48	62.98	3.39E-05
PRR11	19	-2.49	13.05	0.000922
LOC100848433	4	-2.49	100.26	2.17E-07
AURKA	13	-2.49	21.85	1.56E-05
SHCBP1	18	-2.50	40.63	4.02E-05
CCNA2	6	-2.50	50.54	3.90E-05
KIF23	10	-2.50	59.02	3.31E-05
DUSP6	5	-2.51	5.95	0.00499
CCNB2	10	-2.52	19.17	5.24E-05
PMCH	5	-2.52	3.12	0.00119
FOXM1	5	-2.52	16.83	0.000211
SLCO3A1	21	-2.53	6.14	0.0116
AHRR	20	-2.53	1.89	0.00381
CASC5	10	-2.53	48.16	2.95E-05
NUSAP1	10	-2.53	38.42	3.67E-05
ARHGAP11A	10	-2.54	28.24	0.000213
LMNB1	7	-2.54	33.12	4.53E-05
CENPN	18	-2.54	16.80	5.78E-06
ANKRD6	9	-2.54	6.40	0.00187
PRC1	21	-2.54	95.17	1.59E-05
NCAPH	11	-2.54	28.42	2.38E-05
KIF20B	26	-2.55	24.52	2.29E-05
KIF11	26	-2.55	81.14	6.12E-06
WDFY4	28	-2.56	1.92	0.00211
CDK1	28	-2.56	45.07	1.70E-05
PPL	25	-2.57	21.86	2.37E-09
CCDC85B	29	-2.57	10.97	0.00233
BUB1	11	-2.57	50.26	4.99E-05
RCAN1	1	-2.58	80.49	2.71E-19
LOC508486	25	-2.60	4.50	0.00140
KATNAL2	24	-2.60	1.65	0.00120
DLGAP5	10	-2.60	39.06	1.60E-05
TROAP	5	-2.61	30.64	4.71E-05
KIF15	22	-2.61	25.70	6.39E-05
KIF18B	19	-2.61	18.93	0.000142
PEG10	4	-2.62	7.57	3.85E-05
CDCA2	8	-2.62	30.56	2.29E-05
HJURP	3	-2.62	49.47	4.73E-06
KIAA0101	10	-2.62	30.40	2.79E-06

PM20D2	9	-2.62	2.43	0.000307
PLK1	25	-2.63	37.06	3.85E-05
BUB1B	10	-2.63	56.69	2.95E-05
EFNB2	12	-2.63	1.09	0.00860
SYT16	10	-2.64	1.30	0.00254
PKD2L1	26	-2.65	9.18	3.12E-05
CKS2	8	-2.66	46.39	1.59E-05
B3GALNT1	1	-2.66	11.91	3.27E-06
LOC783804	26	-2.66	6.41	0.000103
CENPE	6	-2.67	52.41	1.81E-05
PSRC1	3	-2.67	5.16	0.000744
NEK2	16	-2.68	10.09	0.000625
CDKN2C	3	-2.69	12.74	3.37E-05
LOC786906	4	-2.70	2.65	0.00167
ESPL1	5	-2.71	60.37	3.64E-05
HS3ST2	25	-2.73	43.38	4.11E-05
LOC100138660	7	-2.73	4.63	0.000675
EPHB2	2	-2.75	4.17	0.00272
GLI1	5	-2.75	2.30	0.00238
PDPN	16	-2.76	1.30	0.0129
CCNF	25	-2.77	32.30	0.000101
KIF2C	3	-2.77	39.71	5.78E-06
FAM83D	13	-2.77	14.68	6.33E-06
E2F2	2	-2.77	1.70	0.00313
UBE2C	13	-2.79	58.03	7.83E-06
CADM3	3	-2.79	3.18	0.000129
MXD3	7	-2.79	9.16	0.000120
SPOCK1	7	-2.80	12.31	0.00305
SPAG5	19	-2.80	49.73	7.01E-06
DEPDC1	3	-2.80	20.11	3.38E-06
CSMD2	3	-2.81	3.46	0.00163
NDC80	24	-2.82	43.06	9.42E-06
LOC100848911	7	-2.82	20.85	7.01E-06
GAS2L3	5	-2.82	11.27	1.45E-05
FAM64A	19	-2.85	26.80	6.23E-05
LRRN4CL	29	-2.86	106.21	4.61E-11
LOC782456	13	-2.86	1.42	0.00122
KIF20A	7	-2.88	80.06	3.37E-06
CENPF	16	-2.89	78.38	1.59E-05
PCDH11Y	X	-2.89	22.45	3.25E-06
LOC512293	4	-2.89	5.34	7.81E-05
DUSP5	26	-2.90	1.04	0.0147
LOC100139916	25	-2.90	1.37	0.0250
MAD2	6	-2.91	32.92	0.0110
CDKN3	10	-2.91	13.07	7.01E-06

E2F8	29	-2.92	19.17	1.17E-05
CERS3	21	-2.97	2.33	0.0137
SLC7A8	10	-2.99	1.25	0.0140
CDCA3	5	-2.99	24.08	2.29E-05
CCNB1	20	-3.00	46.56	1.33E-05
ADAMTSL1	8	-3.02	1.11	0.000372
LOC509513	4	-3.04	1.09	0.0405
CXCR4	2	-3.06	3.52	0.00477
NEFH	17	-3.08	1.62	0.0240
MKI67	26	-3.08	171.08	3.34E-06
LOC100848949	1	-3.09	4.59	0.00103
CDC25B	13	-3.10	31.83	4.51E-07
LOC100299874	9	-3.16	7.55	1.59E-05
CDC20	3	-3.18	43.67	2.29E-06
ITGA4	2	-3.23	1.10	0.0143
ARHGAP18	9	-3.24	14.74	2.87E-07
AQP1	4	-3.28	774.74	6.35E-06
MME	1	-3.28	2.52	0.0231
LOC100847220	X	-3.29	1.16	0.00413
SOD3	6	-3.30	2.22	2.29E-05
TMEM158	22	-3.31	8.18	3.26E-06
ADAMTSL1	8	-3.32	15.32	8.78E-12
MESP2	21	-3.34	1.42	6.64E-05
TLR2	17	-3.46	1.01	3.39E-05
ID1	13	-3.47	22.42	1.20E-10
PDE10A	9	-3.48	4.44	7.76E-05
RAVER2	3	-3.52	1.44	8.57E-06
LOC100848808	4	-3.54	6.24	6.30E-05
SYT17	25	-3.56	1.41	0.000137
ADM	15	-3.57	28.63	2.58E-09
TOP2A	19	-3.60	244.84	3.85E-07
DUOX1	10	-3.67	1.78	0.000377
THBD	13	-3.68	13.17	9.27E-08
XPNPEP2	X	-3.72	22.51	4.28E-08
ASPM	16	-3.73	77.64	1.71E-07
PDE1C	4	-3.75	5.16	0.000584
FOSL1	29	-3.81	34.24	4.93E-07
CTSL1	8	-3.82	5.47	3.85E-13
CENPA	11	-3.89	23.56	1.10E-06
MAP2	2	-3.94	3.51	0.00105
HPGD	8	-3.98	15.41	2.16E-07
LOC100847727	4	-3.98	13.48	2.76E-19
GATM	10	-4.05	2.26	3.96E-07
CXHXorf57	X	-4.17	8.17	3.39E-05
GSG2	19	-4.17	16.85	3.56E-05

LOC782601	27	-4.21	2.49	9.73E-05
LOC781565	1	-4.27	24.04	0.000744
SCN1A	2	-4.28	5.22	4.73E-05
ADAMTSL1	8	-4.34	4.53	1.59E-05
NGEF	3	-4.34	2.64	0.00309
SCN9A	2	-4.41	4.16	1.47E-06
FRMD4B	22	-4.44	1.19	0.00307
COL6A6	1	-4.46	4.59	0.0298
DIRAS3	3	-4.66	1.25	1.45E-07
DKK2	6	-5.15	9.38	1.40E-06
MT2A	18	-5.16	9.53	9.55E-06
COLEC12	24	-5.22	4.89	5.87E-10
PODXL	4	-5.43	3.38	0.00925
LPXN	15	-5.62	1.59	1.80E-11
CXCL12	28	-5.73	18.84	0.000205
EMID1	17	-5.78	1.93	4.73E-05
ESM1	20	-5.87	20.24	0.000700
LOC613534	6	-6.24	43.65	0.000877
EFNA5	7	-6.31	1.32	1.09E-09
MEGF6	16	-6.51	84.52	9.19E-14
TGM3	13	-7.05	4.07	0.000179
RXFP1	17	-7.23	1.34	2.13E-06
FBP1	8	-7.49	1.64	9.15E-12
ANGPTL5	15	-7.89	1.57	0.00267
LOC100848103	25	-7.90	10.08	1.52E-33
KCNG1	13	-8.05	1.04	3.85E-06
PTGS1	11	-8.49	18.84	9.43E-15
F3	3	-9.10	20.19	1.67E-15
LOC530437	12	-9.26	1.12	0.00477
NLGN4Y	X	-9.32	3.01	8.49E-08
CYP27C1	2	-12.92	1.59	9.06E-06
LOC515128	16	-12.97	1.41	0.000202
ANKRD24	7	-13.28	4.40	2.35E-21
ASIP	13	-14.01	17.84	8.24E-34
SLC32A1	13	-16.47	1.22	5.63E-10
MMP3	15	-23.82	1.14	1.79E-06
COBL	4	-26.60	1.36	0.000195
CRISPLD1	14	-29.94	4.56	2.96E-11
LOC100848478	4	-54.57	39.51	5.51E-06
RBM44	3	-152.00	5.89	1.12E-06
PRSS2	4	-580.23	2.51	0.000174

Hour 2

Gene	Chromosome	FC	CPM	FDR
IGSF9B	29	137.66	1.40	4.73E-29
KRT5	5	76.54	1.15	2.98E-05
LOC100848478	4	46.21	40.89	1.07E-05
MAPK13	23	36.32	1.51	3.28E-33
PAX2	26	26.42	1.05	8.82E-12
FAM150A	14	13.05	1.13	0.00143
AFAP1L2	26	12.14	3.46	6.29E-07
MATN3	11	11.22	12.87	0.0150
CD200	1	10.47	2.11	3.77E-07
PIK3IP1	17	7.73	2.65	3.64E-06
ISLR2	21	7.48	2.21	3.84E-08
GDAP1L1	13	7.48	1.46	0.000134
LOC615559	21	6.95	1.26	0.000278
TNF	23	6.90	1.73	0.00628
DSC2	24	6.84	16.45	0.000620
HEYL	3	6.44	10.90	0.00156
GLRB	17	6.16	2.38	2.79E-05
USP43	19	5.97	2.80	5.30E-07
TNFSF18	16	5.95	24.33	7.96E-15
CFH	16	5.41	14.86	0.000372
RXFP4	3	5.40	1.74	6.83E-11
C28H10orf10	28	5.26	1.22	0.000576
LOC100336535	19	5.18	2.43	1.15E-06
MEST	4	5.07	4.00	5.01E-05
GALNTL2	1	4.95	5.36	3.10E-10
EPOR	7	4.80	1.28	0.000227
ACAN	21	4.78	50.74	0.000650
LAMB3	16	4.68	2.50	1.41E-11
RASGEF1B	6	4.63	1.10	0.00772
GPR133	17	4.51	4.23	1.71E-05
WNT2B	3	4.48	1.42	0.0362
DSC3	24	4.42	47.85	0.00992
TPPP	20	4.40	1.54	5.02E-07
TMEM88	19	4.37	2.34	1.57E-08
LOC100847471	8	4.35	1.41	6.29E-07
WDR35	11	4.27	54.49	2.56E-17
EGR3	8	4.26	11.47	8.58E-14
PEAR1	3	4.23	5.19	2.63E-09
SLC6A6	22	4.17	14.57	7.95E-08
ABCA1	8	4.11	6.89	4.06E-06
LOC100336584	24	4.11	1.17	0.000165
HES4	16	4.10	4.13	2.87E-05

GAS6	12	4.05	6.26	1.79E-13
LIF	17	4.05	58.70	0.00105
TLR4	8	4.03	2.04	2.38E-05
LOC781004	16	4.03	1.27	0.000635
SEMA5B	1	4.02	40.50	1.26E-13
FAM131B	4	3.98	3.12	1.72E-08
C18H19orf33	18	3.96	1.63	2.49E-07
ATP8B1	24	3.95	44.34	0.0115
RASSF5	16	3.92	6.62	0.0105
GIPC3	7	3.90	5.81	3.41E-06
ICOSLG	1	3.86	1.80	0.00939
TARSL2	21	3.80	1.34	0.0235
TBX2	19	3.75	1.88	0.000966
SEPT4	19	3.74	2.09	0.000192
IGFBP7	6	3.74	25.25	0.00104
HEY1	14	3.70	3.97	0.000339
GRIP2	22	3.65	1.21	0.00124
LOC539821	16	3.65	1.13	3.47E-06
IGF2BP2	1	3.61	4.01	0.00350
HAS1	18	3.60	2.63	8.19E-06
MGP	5	3.60	85.11	1.89E-05
C26H10orf92	26	3.59	1.09	2.30E-06
SEMA5A	20	3.56	3.44	0.000142
ODZ4	29	3.54	6.26	0.000368
MGC148692	6	3.54	1.84	4.62E-07
COL11A1	3	3.54	842.39	0.000112
COL8A1	1	3.53	9.67	0.0145
KRBA1	4	3.53	1.64	8.22E-06
LOC100336868	16	3.48	1.91	0.00131
MGAT5B	19	3.47	1.54	0.0453
CARD11	25	3.46	2.16	0.0475
LOC535166	14	3.45	4.76	0.000701
GREB1	11	3.44	1.76	0.0335
LMX1B	11	3.44	1.03	0.0302
PPP1R14A	18	3.42	1.46	0.000218
TREH	15	3.40	1.15	2.33E-05
JAKMIP3	26	3.39	1.75	2.56E-06
LOC506672	2	3.39	3.74	0.0214
FAM83H	14	3.36	7.01	9.17E-05
RASGRP2	29	3.36	5.46	0.000173
LOC100848636	5	3.34	2.81	0.00492
ZFHX4	14	3.31	9.66	2.44E-05
CSRNP1	22	3.30	23.64	8.48E-10
BCAS1	13	3.30	3.56	0.0155
FOSB	18	3.29	56.94	2.03E-13

BBC3	18	3.26	2.85	3.84E-08
TCP11L2	5	3.26	5.19	0.00650
LOC100848128	11	3.25	2.65	0.00133
SYN2	22	3.21	1.57	0.000197
CA11	18	3.17	1.12	0.0227
DMPK	18	3.17	10.16	7.13E-15
NPDC1	11	3.16	5.32	0.000694
ANKH	20	3.15	136.66	1.69E-14
TSPAN2	3	3.14	1.52	0.00156
BNC2	8	3.14	4.23	9.76E-05
GPX3	7	3.14	19.93	0.00422
OLFM2	7	3.11	9.11	5.26E-05
SPEG	2	3.11	12.38	3.06E-12
PNPLA1	23	3.09	1.57	0.000196
PODN	3	3.08	2.20	0.0498
FGF10	20	3.06	11.59	0.00831
OSCAR	18	3.06	1.98	0.00211
SLC25A13	4	3.05	4.42	0.00221
SPINT2	18	3.05	18.53	4.64E-16
HIST1H1C	23	3.03	2.30	0.00407
B4GALNT1	5	3.03	1.67	0.000122
LOXL3	11	3.03	17.36	3.43E-08
DNAH2	19	3.03	2.54	2.63E-05
ABCA6	19	3.02	5.11	0.0422
FGF19	29	3.01	3.03	0.0145
GDAP1	14	3.01	3.22	0.00256
LOC613534	6	3.00	28.12	0.0122
LOC100847959	16	3.00	1.28	3.20E-05
CISH	22	2.99	1.69	0.000195
ITGA11	10	2.99	197.53	4.64E-06
CABLES1	24	2.98	8.60	2.19E-05
KDM6B	19	2.97	74.88	2.35E-08
CATSPERG	18	2.96	13.74	4.78E-12
SLC13A4	4	2.96	2.46	0.0458
LOC100848739	14	2.94	4.39	6.58E-09
TGFB2	16	2.94	45.14	9.08E-08
PM20D1	16	2.93	6.97	0.000182
TET3	11	2.92	2.44	0.000192
TCEA3	2	2.89	1.23	0.00548
ATP6AP1L	7	2.89	3.96	2.38E-05
RDH10	14	2.86	36.22	1.78E-07
GDF15	7	2.86	1.43	0.00266
RAB15	10	2.84	1.33	0.00105
CASQ2	3	2.83	2.18	0.0151
GPR132	21	2.81	1.74	0.00317

HIPK4	18	2.81	1.09	0.000126
TRPV4	17	2.81	3.25	0.000796
KCNK6	18	2.76	17.34	1.23E-07
CCL26	25	2.75	2.32	0.0131
C20H5orf41	20	2.74	8.81	5.50E-06
LOC100847602	5	2.72	12.77	2.35E-05
RHOD	29	2.72	3.88	0.000350
KLHL24	1	2.71	4.88	4.45E-05
CHRD	1	2.69	6.39	6.09E-05
STX1A	25	2.69	2.38	7.15E-05
TOB2	5	2.69	34.30	7.83E-16
RHBDL2	3	2.67	2.84	0.000252
YPEL2	19	2.67	6.90	6.84E-06
AGRN	16	2.66	1.58	4.75E-05
SUSD4	16	2.66	1.10	0.0376
RASGRF2	7	2.66	3.51	0.00297
ZMYND15	19	2.65	4.19	0.00570
RAB11FIP1	27	2.65	5.42	0.00203
SLC6A16	18	2.64	3.00	0.000252
PPP1R42	14	2.62	1.34	0.0110
PCDH1	7	2.61	1.33	0.000993
OBSL1	2	2.60	12.29	0.000162
PROS1	1	2.60	105.99	0.0221
AQP11	29	2.60	1.53	0.00389
PIM1	23	2.60	43.56	9.35E-07
UNC5B	28	2.59	7.76	3.43E-05
CELSR3	22	2.59	1.31	0.000380
IER2	7	2.59	27.17	2.36E-13
GPSM3	23	2.58	1.00	0.0200
FGD6	5	2.58	3.63	0.000155
TGFB3	10	2.58	31.26	1.70E-10
FAM115C	4	2.57	3.23	0.00691
ALDOC	19	2.57	15.43	0.000133
CCNG2	6	2.56	4.48	8.93E-05
GATA2	22	2.56	9.32	0.00221
ARC	14	2.56	2.55	0.00544
LOC100296463	25	2.54	11.28	1.16E-07
STRA6	21	2.54	1.44	0.0285
IRF1	7	2.54	45.11	0.0176
LOC100847766	X	2.54	1.40	0.0109
LOC100847829	X	2.54	1.40	0.0109
SLC29A2	29	2.53	6.46	0.000176
JUNB	7	2.53	103.99	3.39E-07
LOC777601	X	2.53	1.40	0.0111
HSF4	18	2.53	6.57	0.00409

IGFBP3	4	2.52	92.54	0.00160
LMTK3	18	2.51	2.49	0.0181
B3GALT2	16	2.51	2.51	0.0272
LOC789485	7	2.51	96.28	0.000950
HES1	1	2.50	18.76	1.67E-08
HSPB6	18	2.49	99.82	1.79E-10
FNDC4	11	2.48	6.29	4.77E-06
MIDN	7	2.47	25.38	1.29E-14
LOC100848038	7	2.46	99.41	0.0257
LOC100848191	18	2.44	96.70	7.69E-11
SPON1	15	2.44	30.79	2.12E-05
IRF5	4	2.43	7.03	0.0184
SDC1	11	2.42	65.99	2.82E-08
NDRG2	10	2.42	3.59	0.0372
NOD1	4	2.42	1.07	0.00262
SLC25A34	16	2.42	3.12	0.00192
DOCK10	2	2.41	30.11	9.77E-07
ITGBL1	12	2.41	19.98	5.95E-05
TNFRSF19	12	2.41	1.41	0.0180
CHCHD6	22	2.41	1.25	0.000741
FRY	12	2.40	16.80	2.67E-08
ATF3	16	2.40	14.97	0.00737
TRAF1	8	2.40	1.84	0.00165
FILIP1L	1	2.39	20.28	0.0405
LOC509006	7	2.38	55.70	9.58E-14
CGN	3	2.37	2.11	0.0190
MNT	19	2.37	7.19	2.42E-06
MMP15	18	2.36	1.66	0.0218
CASK	X	2.35	114.70	4.90E-05
NFKBIA	21	2.35	135.46	0.00721
FAM105A	20	2.35	2.42	0.0318
ZC3H6	11	2.34	1.95	0.000918
LOC100847694	X	2.33	1.76	0.0335
UNC5C	6	2.33	8.65	0.0292
LOC784236	8	2.33	1.14	0.00467
CBFA2T3	18	2.32	17.17	0.000155
C8H9orf103	8	2.32	2.01	0.00574
CSDC2	5	2.32	1.16	0.0108
CACNA1A	7	2.31	13.70	0.00620
TBC1D16	19	2.31	10.49	0.000216
PRICKLE1	5	2.31	2.54	0.000270
LOC100847795	5	2.30	1.97	0.000138
SARM1	19	2.30	3.64	0.00888
C15H11orf96	15	2.30	66.07	0.00546
ST8SIA1	5	2.30	9.82	0.00120

MAFK	25	2.30	8.24	1.31E-05
GPR63	9	2.29	9.33	0.00128
SPTBN2	29	2.29	3.96	0.0110
MICALL2	25	2.29	6.34	4.60E-06
BCL3	18	2.28	33.78	6.95E-05
CHPF	2	2.28	123.48	2.62E-08
SCG5	10	2.27	8.43	1.84E-05
TNFRSF10A	8	2.27	10.46	0.00305
PRSS48	17	2.27	1.20	0.0231
VWA5A	29	2.26	6.18	0.0342
LOC100299061	X	2.26	1.56	0.0164
FN1	2	2.26	11020.61	0.0158
ZNF462	8	2.25	9.48	1.08E-05
LOC100848206	X	2.25	3.94	0.00535
NHS	X	2.25	5.19	4.66E-05
FAM198B	17	2.25	5.41	0.000816
FBXO32	14	2.25	3.89	5.62E-05
EFEMP1	11	2.24	75.42	0.00801
NFKBIZ	1	2.24	94.35	0.0301
TNFRSF4	16	2.24	1.85	0.0157
IER3	23	2.23	144.29	0.000900
B3GNT8	18	2.23	2.54	0.00229
DTNA	24	2.22	3.70	0.0113
C29H11orf80	29	2.22	9.39	0.000120
LOC100137797	18	2.22	2.22	0.00709
CDH17	14	2.22	1.43	0.00410
NMRAL1	25	2.21	7.14	0.00178
PRUNE2	8	2.21	15.78	0.000694
B3GNT2	11	2.21	33.11	3.23E-06
ARRDC2	7	2.21	19.22	0.00576
KLF10	14	2.21	92.72	2.92E-11
NOXA1	11	2.21	2.90	0.000554
MAPK12	5	2.20	2.76	0.00998
CDC42BPG	29	2.20	12.18	2.60E-06
ENOX1	12	2.19	5.66	0.0164
ARHGEF5	4	2.19	8.02	0.00514
NFKBID	18	2.19	8.75	0.000617
IL34	18	2.19	7.24	1.87E-05
SEMA4A	3	2.18	5.11	0.00523
CNNM2	26	2.18	28.19	0.00126
SEMA4F	11	2.18	4.68	4.37E-05
ADAMTS16	20	2.18	1.46	0.0104
GREB1L	24	2.17	6.95	0.00427
FGD4	5	2.17	3.84	0.000142
LOC100848155	3	2.17	1.71	0.00536

MRVI1	15	2.17	20.55	3.58E-06
HSD11B1L	7	2.17	3.13	0.000193
DBP	18	2.17	2.61	0.00352
SLC43A2	19	2.17	3.73	0.00269
C10H14orf93	10	2.17	1.86	0.00385
LOC786974	20	2.16	8.58	1.56E-05
CYP1B1	11	2.16	35.85	0.00747
KLHL32	9	2.16	5.22	0.00192
EXD1	10	2.16	3.77	0.0148
VWF	5	2.15	1.51	0.0308
LOC100847427	1	2.15	14.01	7.48E-06
C22H3orf62	22	2.14	2.52	0.00352
ARHGAP24	6	2.14	4.67	0.0291
FAP	2	2.14	59.98	1.65E-05
VWCE	29	2.14	1.66	0.0407
STOM	8	2.13	159.12	7.90E-05
GDAP2	3	2.13	11.47	0.0453
SYNPO2	6	2.13	176.96	0.00315
LOC100294865	22	2.13	61.45	2.68E-12
CORO2B	10	2.13	14.98	9.05E-06
CAPRIN2	5	2.12	8.25	0.00221
TMC6	19	2.12	25.39	2.44E-08
YPEL3	25	2.11	6.27	0.00124
NKX2-2	13	2.11	1.75	0.0272
ADSSL1	21	2.11	1.74	0.00369
LOC100848152	19	2.10	8.10	0.0148
MXD1	11	2.10	6.43	0.00967
OLFML2B	3	2.10	45.43	0.000266
COL4A2	12	2.10	494.20	1.19E-05
HOXC13	5	2.10	2.72	0.00889
CPT1C	18	2.09	1.28	0.0117
JAG1	13	2.08	206.72	0.000102
AK7	21	2.08	1.24	0.0113
FOXO3	9	2.08	4.14	0.00185
ARMCX1	X	2.08	3.44	0.00260
PTPRH	18	2.07	1.90	0.0177
ID2	11	2.07	75.66	0.000462
P4HA3	15	2.07	9.81	0.00522
PNRC1	9	2.06	6.79	0.000234
ITPKC	18	2.06	11.56	3.78E-05
MOSC2	16	2.06	3.73	0.00292
LOC788414	2	2.06	1.44	0.0362
ELOVL7	20	2.06	20.76	0.0279
CST3	13	2.05	97.01	2.18E-05
LOC100335357	12	2.05	2.37	0.000944

LRRC20	28	2.05	3.56	0.000648
TMEM146	7	2.05	12.93	2.57E-06
SLC38A3	22	2.05	1.01	0.0219
ZMAT3	1	2.05	14.65	2.03E-05
ZBTB46	13	2.05	2.62	0.00221
APOE	18	2.05	9.81	0.0166
SMAD3	10	2.05	33.87	1.69E-07
NUAK2	16	2.05	45.44	1.44E-10
CTSZ	13	2.04	67.93	1.35E-06
CALCRL	2	2.04	10.56	0.0192
BHLHE40	22	2.04	434.04	2.24E-05
GADD45B	7	2.04	75.12	3.43E-08
SOX13	16	2.03	1.46	0.0318
HHIPL1	21	2.03	6.91	0.00251
AGMO	4	2.03	28.82	1.01E-08
ZSWIM4	7	2.03	22.11	6.79E-06
PTER	13	2.03	1.86	0.0180
AGPAT4	9	2.03	8.06	0.00968
FAM46B	2	2.03	3.00	0.00683
RND2	19	2.03	1.46	0.00593
WNT10B	5	2.03	13.58	0.00609
LOC100848220	12	2.03	1.63	0.0154
IL11	18	2.02	2.12	0.0272
PER1	19	2.02	5.45	0.000785
C25H16orf71	25	2.02	1.75	0.0362
ACTC1	10	2.01	1.26	0.0324
PNPLA2	29	2.01	9.06	0.000201
ABHD4	10	2.01	10.38	0.00225
LRFN3	18	2.01	1.64	0.0190
SEMA3A	4	2.01	8.87	1.44E-05
DOCK11	X	2.00	5.91	0.0430
MET	4	-2.00	16.98	1.46E-06
MELK	8	-2.00	23.10	0.00244
LOC787564	15	-2.00	1.61	0.0200
EZH2	4	-2.01	51.26	0.000162
PROM1	6	-2.01	5.79	0.0420
SLC2A1	3	-2.01	35.09	1.26E-05
WDFY4	28	-2.01	1.27	0.0147
ROBO2	1	-2.01	9.50	0.0220
MCM5	5	-2.01	58.07	0.00300
FANCI	21	-2.01	36.41	0.000633
TGFA	11	-2.01	3.13	0.0212
APITD1	16	-2.01	16.06	0.000634
LOC100847825	1	-2.02	2.16	0.0124
NTN1	19	-2.02	21.16	0.00288

LOC100848655	5	-2.03	16.60	0.000120
TMPO	5	-2.03	63.14	0.000291
SMC2	8	-2.03	79.40	0.000351
SGOL2	2	-2.03	20.35	0.000304
INCENP	29	-2.03	40.08	0.000396
CASC5	10	-2.03	38.10	0.00281
TYMS	24	-2.03	48.71	0.000179
MREG	2	-2.04	3.89	0.0235
CCNB1IP1	10	-2.04	3.93	0.00274
TRIP13	20	-2.04	29.79	0.000477
UHRF1	7	-2.04	47.54	0.000648
FAM72A	16	-2.05	5.69	0.00542
KIF26B	16	-2.05	31.24	0.0296
PSD2	7	-2.05	3.66	0.0139
FAM54A	9	-2.05	17.90	0.000136
GAS1	8	-2.05	2.81	0.00790
H2AFZ	6	-2.05	119.87	0.000122
CTNNAL1	8	-2.05	71.84	5.75E-11
LOC526200	9	-2.05	84.82	1.16E-06
MCM2	22	-2.06	56.10	0.000650
PBK	8	-2.06	59.20	0.000217
ESCO2	8	-2.06	12.73	0.00390
DTYMK	3	-2.07	19.49	0.00401
DIAPH3	12	-2.07	83.65	0.000362
LOC513969	13	-2.08	1.86	0.00370
TGFBR3	3	-2.08	27.18	0.000162
RBM8A	3	-2.08	27.80	0.00279
TNFRSF6B	13	-2.08	17.79	0.0177
CLSPN	3	-2.08	17.59	0.00771
PCOLCE2	1	-2.09	6.99	0.00739
SLCO3A1	21	-2.09	5.76	0.0453
DEPDC1B	20	-2.09	6.49	0.00519
DUT	10	-2.09	39.35	5.57E-05
ESRRG	16	-2.10	3.56	0.00709
LRR1	10	-2.10	4.54	0.00238
SELRC1	3	-2.10	4.58	0.000462
RRM2	11	-2.10	206.82	0.00136
TTK	9	-2.11	4.03	0.0117
HMMR	7	-2.11	41.93	0.000352
RNASEH2A	7	-2.11	40.11	0.000991
WHSC1	6	-2.11	51.07	0.000641
MCM3	23	-2.11	57.39	0.000414
MASP1	1	-2.12	31.10	0.0151
SDK2	19	-2.12	8.97	0.0123
LOC100847721	9	-2.12	15.12	0.00185

PCDH11Y	X	-2.13	18.51	0.00237
NXT1	13	-2.13	9.83	0.00124
CEP72	20	-2.13	8.33	0.0155
OSR2	14	-2.13	3.70	0.000577
PRR11	19	-2.13	9.14	0.0104
KIF23	10	-2.13	48.83	0.00129
CKS1B	3	-2.15	21.01	0.000462
CENPT	18	-2.15	22.45	0.00204
COL27A1	8	-2.15	10.68	1.44E-05
LOC100847964	28	-2.16	3.44	0.00121
CENPK	20	-2.16	17.73	0.000119
RND3	2	-2.16	80.86	1.01E-10
CENPQ	23	-2.16	16.01	0.000431
KIF15	22	-2.16	19.23	0.00186
PTGR1	8	-2.16	13.90	0.00717
LOC786705	21	-2.17	1.72	0.00203
LOC100848488	1	-2.17	1.08	0.0422
C5H12orf48	5	-2.17	9.78	0.000252
SH3BP2	6	-2.18	37.72	5.36E-06
PMF1	3	-2.19	10.35	0.00104
TSPAN13	4	-2.19	46.33	5.55E-14
CACNA1C	5	-2.20	3.75	0.00346
CENPE	6	-2.20	43.31	0.000131
CCDC99	20	-2.21	29.54	0.000120
DRD3	1	-2.21	1.15	0.0136
CENPF	16	-2.21	54.76	0.00219
ERCC6L	X	-2.21	7.88	0.00297
FOXM1	5	-2.22	11.80	0.00521
ASF1B	7	-2.22	13.88	0.00229
IGFBP4	19	-2.22	410.26	0.00253
NDC80	24	-2.22	31.56	0.000487
KIF20B	26	-2.22	15.80	0.00108
PSMB8	23	-2.23	2.78	0.00132
SPC25	2	-2.23	10.08	0.000264
SPHK1	19	-2.23	65.28	1.01E-06
AURKB	19	-2.23	25.98	0.000168
CCBE1	24	-2.23	8.55	0.00149
CCDC34	15	-2.24	7.48	0.000889
RAD51AP1	5	-2.24	12.61	0.000640
PLXNA2	16	-2.24	9.64	0.000445
NUSAP1	10	-2.24	32.73	0.000195
LOC788599	18	-2.25	1.01	0.0287
OIP5	10	-2.25	9.30	0.00200
STMN1	2	-2.25	62.12	0.000106
NCAPD2	5	-2.25	60.37	0.000252

C10H15orf23	10	-2.26	13.26	0.000312
CMIP	18	-2.26	54.66	1.09E-07
SLC7A8	10	-2.26	1.12	0.00559
LRIG3	5	-2.26	12.10	1.80E-05
CEP55	26	-2.26	25.48	0.000306
BHLHE41	5	-2.27	1.51	0.000749
HMGB3	X	-2.27	2.82	0.0122
E2F8	29	-2.27	10.57	0.00349
CCNF	25	-2.28	21.50	0.00465
NUF2	3	-2.28	34.83	5.33E-05
CKAP2L	11	-2.29	20.23	0.000262
KIF11	26	-2.30	73.42	2.81E-05
HJURP	3	-2.30	31.64	0.000441
LOC100849043	20	-2.30	1.56	0.00335
ODZ2	7	-2.32	2.42	0.00144
KPNA2	19	-2.32	190.31	4.03E-05
MYBL2	13	-2.32	87.32	0.000237
SMC4	1	-2.32	107.26	6.84E-06
DLGAP5	10	-2.33	33.90	0.000164
AURKA	13	-2.33	20.04	0.000157
MXD3	7	-2.33	5.64	0.0217
LOC100336690	5	-2.33	18.72	0.000139
LOC614357	5	-2.34	1.92	0.00540
CEP120	7	-2.35	1509.62	0.0281
NFATC2	13	-2.35	4.25	0.00177
DNM1	11	-2.35	18.74	1.44E-07
NDP	X	-2.35	1.65	0.0194
SKA3	12	-2.35	20.75	1.19E-05
CDCA2	8	-2.35	24.60	4.66E-05
LOC100848433	4	-2.35	94.44	1.12E-06
CADM3	3	-2.36	3.20	0.00108
CSMD2	3	-2.36	3.35	0.00268
LOC100848011	22	-2.37	3.64	0.000336
LSAMP	1	-2.37	132.92	0.0278
SKA1	24	-2.37	10.96	2.40E-05
SHCBP1	18	-2.37	40.13	4.75E-05
GTSE1	5	-2.37	28.59	0.000252
LOC100337116	X	-2.37	264.24	0.0181
CCNA2	6	-2.37	48.24	0.000130
PPL	25	-2.38	17.67	9.51E-09
PMCH	5	-2.38	2.63	0.00174
ANLN	4	-2.38	283.68	1.13E-05
LOC100138660	7	-2.39	4.52	0.00241
LMNB2	7	-2.39	20.62	7.02E-05
KIFC1	23	-2.39	48.56	0.000445

BUB1	11	-2.40	42.05	0.000164
CKAP2	12	-2.40	115.13	7.27E-05
PEG10	4	-2.40	6.38	0.000447
NCAPH	11	-2.41	25.39	5.69E-05
KIAA0101	10	-2.41	26.94	2.16E-05
LOC100337426	3	-2.42	39.54	0.0454
RGS17	9	-2.42	10.93	8.47E-05
CENPN	18	-2.42	17.71	1.78E-05
LOC509810	18	-2.42	4.09	0.000235
LOC618307	3	-2.42	1.13	0.00786
ANKRD6	9	-2.42	4.54	0.0128
LOC100848726	29	-2.44	12.65	0.00540
CERS3	21	-2.44	2.33	0.0480
KIF22	25	-2.44	47.59	1.65E-05
CADM1	15	-2.44	5.33	3.43E-05
TACC3	6	-2.45	56.00	8.55E-05
B3GALNT1	1	-2.45	9.14	8.89E-06
CDKN2C	3	-2.46	10.02	0.000362
BIRC5	19	-2.47	40.85	4.63E-05
DBF4B	19	-2.47	3.08	0.0235
CDCA8	3	-2.47	27.54	2.57E-05
ARHGAP18	9	-2.47	11.17	7.52E-05
MAD2L1	6	-2.48	28.79	7.43E-06
BUB1B	10	-2.48	42.36	0.000362
NCAPD2	5	-2.48	1.77	0.0123
CDK1	28	-2.51	43.10	2.56E-05
FBXO5	9	-2.51	8.75	4.71E-05
ESPL1	5	-2.52	53.22	0.000155
EXO1	16	-2.52	7.01	0.000257
PIR	X	-2.53	6.60	2.10E-05
PKD2L1	26	-2.53	10.15	0.000157
FABP3	2	-2.53	10.62	5.01E-05
LOC787405	10	-2.54	3.65	0.0404
FIGNL1	4	-2.54	5.30	0.000342
LMNB1	7	-2.54	32.71	1.31E-05
PRC1	21	-2.56	92.59	2.19E-05
DEPDC1	3	-2.56	17.09	3.41E-06
SGOL1	1	-2.56	7.89	0.000414
SGK1	9	-2.57	51.90	2.71E-10
LOC100336476	3	-2.59	3.13	0.0102
CKS2	8	-2.59	47.34	9.05E-06
ARHGAP11A	10	-2.59	24.14	0.000312
CH25H	26	-2.59	23.23	5.74E-06
WNT5A	22	-2.59	21.61	0.00194
SPAG5	19	-2.60	45.26	9.17E-05

TROAP	5	-2.63	30.46	0.000221
EFNB2	12	-2.63	2.57	0.0143
KIF2C	3	-2.63	36.87	1.49E-05
C19H17orf53	19	-2.63	2.54	0.0147
C11H2orf40	11	-2.63	2.81	0.0482
GAS2L3	5	-2.63	7.46	0.000755
RAVER2	3	-2.64	1.05	0.00349
LOC783804	26	-2.64	7.00	0.000262
LOC786906	4	-2.66	2.32	0.00124
MAN1C1	2	-2.67	5.99	0.00286
NPAS2	11	-2.67	4.83	3.27E-06
CCNB2	10	-2.68	18.61	4.75E-05
HMOX1	5	-2.69	62.67	2.03E-13
MT2A	18	-2.70	15.70	0.00557
PLK1	25	-2.71	32.52	5.46E-05
LBH	11	-2.71	12.12	0.00757
SV2C	10	-2.71	3.87	1.15E-06
LOC508486	25	-2.71	4.15	0.00103
TRAIP	22	-2.73	2.73	0.0146
LOC100299874	9	-2.76	6.46	0.000421
MKI67	26	-2.76	159.88	5.09E-05
ZMIZ1	28	-2.76	59.31	1.29E-18
CDKN3	10	-2.78	9.81	0.000133
FAM83D	13	-2.79	10.54	7.67E-06
SYT16	10	-2.81	1.23	0.00237
TLR2	17	-2.81	1.80	0.000111
NEK2	16	-2.82	7.89	5.79E-05
FAM110A	13	-2.84	1.98	0.00216
SPOCK1	7	-2.87	12.59	0.00233
CDCA3	5	-2.88	22.95	8.92E-05
AQP1	4	-2.88	763.62	7.78E-05
LOC100848949	1	-2.89	4.71	0.00195
ADAMTSL1	8	-2.90	4.41	9.19E-05
HS3ST2	25	-2.91	87.02	2.81E-06
LOC512293	4	-2.92	5.23	0.000266
KIF20A	7	-2.96	76.76	1.31E-05
UBE2C	13	-2.97	64.06	1.41E-05
LOC100848911	7	-2.97	20.45	4.23E-06
SERPINB2	24	-2.97	10.04	0.0105
CDC20	3	-2.98	37.41	1.67E-05
PSRC1	3	-2.99	2.50	0.00989
EPHB2	2	-3.00	4.04	0.00531
SLC37A1	1	-3.02	1.05	0.0270
CDC25B	13	-3.04	27.15	1.31E-05
KIF18B	19	-3.04	14.73	4.63E-05

ZNF280C	X	-3.06	1.46	3.21E-06
FAM64A	19	-3.06	29.10	9.19E-05
ADAMTSL1	8	-3.09	14.28	5.08E-08
LRRN4CL	29	-3.12	118.18	2.87E-10
CCNB1	20	-3.12	45.75	4.79E-06
ASPM	16	-3.12	62.61	6.11E-06
RASL11B	6	-3.19	3.52	3.34E-09
MAD2	6	-3.19	29.86	0.00503
TOP2A	19	-3.21	197.19	1.44E-05
TBC1D25	X	-3.22	12.05	0.0465
LOC615206	26	-3.23	2.92	0.000549
PDE1C	4	-3.26	4.16	0.00166
LOC100847220	X	-3.32	1.11	0.000802
LOC511531	3	-3.35	2.03	0.00122
SCN9A	2	-3.37	2.54	0.00195
LOC100848808	4	-3.47	10.57	1.13E-10
XPNPEP2	X	-3.48	20.09	1.82E-07
TMEM158	22	-3.53	17.37	2.82E-10
DUOX1	10	-3.54	1.45	0.000414
LOC100847727	4	-3.64	12.01	1.14E-16
LOC100847497	X	-3.65	1.16	3.43E-05
PM20D2	9	-3.66	1.63	4.03E-06
CENPA	11	-3.71	24.25	1.96E-07
TMEM100	19	-3.72	1.62	0.00111
RXFP1	17	-3.77	1.13	0.000526
CTSL1	8	-3.79	4.83	4.72E-12
SYT17	25	-3.80	1.11	0.000383
SOD3	6	-3.81	2.17	1.65E-05
HPGD	8	-3.89	13.85	1.83E-07
JAG2	21	-3.90	1.87	0.00104
NGEF	3	-3.91	2.63	0.00105
CXHXorf57	X	-3.92	5.88	1.95E-05
LOC100139916	25	-4.15	1.11	0.00139
THBD	13	-4.17	21.34	2.30E-12
MESP2	21	-4.22	1.48	2.87E-07
LOC100336526	2	-4.23	1.04	2.07E-05
GATM	10	-4.33	1.55	2.45E-05
GSG2	19	-4.33	15.88	3.93E-05
MTSS1	14	-4.41	2.32	1.53E-12
GLI1	5	-4.50	2.47	7.19E-07
SCN1A	2	-4.50	4.08	5.04E-06
DIRAS3	3	-4.63	1.37	8.76E-08
LOC782601	27	-4.67	2.52	7.00E-06
ESM1	20	-4.71	35.27	1.28E-07
ADM	15	-4.78	34.42	2.43E-21

RPS27	17	-4.83	12.51	0.0233
EFNA5	7	-4.96	1.10	8.66E-07
MMP27	15	-4.96	1.08	0.000107
DKK2	6	-5.32	9.45	1.39E-08
ADRB2	7	-5.49	7.80	2.85E-10
TGM3	13	-5.50	4.32	4.77E-09
EMID1	17	-5.52	1.87	2.61E-05
COLEC12	24	-5.61	5.55	2.17E-11
F3	3	-5.71	257.33	6.84E-21
LOC518986	19	-5.85	4.37	9.76E-10
MEGF6	16	-5.90	88.82	5.85E-11
AJAP1	16	-6.05	2.23	4.38E-09
PDE10A	9	-6.76	6.05	2.63E-09
FRMD4B	22	-7.00	1.08	0.00110
CXCL12	28	-7.36	20.23	4.05E-06
NLGN4Y	X	-8.64	2.51	8.60E-07
ANKRD24	7	-9.06	4.08	8.75E-11
LOC100848103	25	-9.46	17.33	8.85E-31
KCNG1	13	-9.58	2.12	4.29E-11
FBP1	8	-9.88	1.49	3.98E-15
PTGS1	11	-9.93	18.38	6.54E-16
LOC515128	16	-10.44	1.29	0.00141
CYP27C1	2	-13.09	1.35	1.79E-08
CRISPLD1	14	-14.02	4.24	9.47E-07
ASIP	13	-15.19	17.29	2.50E-48
SLC32A1	13	-15.84	1.75	2.36E-13
LOC100336748	9	-16.23	2.12	1.38E-05
LOC100296277	X	-40.90	6.46	0.00157
COBL	4	-45.64	1.66	5.51E-06
RBM44	3	-195.78	4.47	8.05E-06
PRSS2	4	-3336.55	2.68	1.52E-05

Hour 8

**Gene**	**Chromosome**	**FC**	**CPM**	**FDR**
KRT17	19	340.73	1.37	0.000274
KRT5	5	91.38	1.53	2.55E-05
LOC100848478	4	61.08	59.28	1.32E-10
PAX2	26	15.87	1.18	1.72E-10
CX3CL1	18	14.77	1.77	8.90E-06
MX2	1	13.96	3.07	1.10E-14
CD200	1	13.00	3.73	9.06E-11
HEYL	3	12.63	3.72	6.45E-05
AFAP1L2	26	12.52	1.98	3.30E-11
MATN3	11	12.22	9.67	0.00518
TBX2	19	10.66	1.02	4.24E-05

RNF122	27	10.46	2.11	0.0442
ICOSLG	1	9.68	1.92	7.93E-06
ISLR2	21	9.51	1.92	3.31E-10
NOS2	19	9.46	1.08	0.000545
IFI27	21	9.41	3.89	5.27E-05
STRA6	21	8.99	7.11	4.54E-12
CCL20	2	8.61	5.15	0.00587
TGM2	13	8.21	1.27	1.05E-15
HES4	16	7.49	1.41	3.01E-07
MAPK13	23	7.33	1.41	0.00151
FAM131B	4	6.83	2.21	9.43E-10
DSC2	24	6.76	13.49	0.00298
RBPMS2	10	6.45	1.24	0.00125
CCL5	19	5.71	52.62	0.000139
GIPC3	7	5.53	4.82	5.15E-10
CFH	16	5.52	14.34	6.89E-05
PAQR7	2	5.43	2.35	5.03E-10
LOC613534	6	5.38	45.66	3.90E-06
CDA	2	5.36	2.53	1.04E-08
GDAP1L1	13	5.34	1.03	0.0162
LIF	17	5.32	6.47	4.87E-05
SLC6A6	22	5.25	8.51	6.00E-05
DSC3	24	5.20	36.66	0.0129
ACAN	21	5.20	39.86	0.000191
FUT1	18	5.14	1.71	1.87E-06
TSPAN2	3	5.13	2.19	1.07E-05
TNFSF18	16	5.11	50.02	1.94E-10
MEST	4	5.09	3.28	0.00991
OLR1	5	4.92	136.46	1.19E-05
AGRN	16	4.75	2.83	4.57E-08
RSAD2	11	4.71	2.23	0.000107
GPR133	17	4.62	2.77	7.36E-05
GLRB	17	4.55	2.15	9.96E-06
IL8	6	4.54	54.29	2.21E-05
PPP1R14A	18	4.54	1.69	6.92E-08
ZBP1	13	4.46	2.77	0.00126
ABCA1	8	4.45	7.66	0.000137
RASSF4	28	4.41	9.87	1.19E-06
VAT1L	18	4.33	1.72	0.0311
C18H19orf33	18	4.33	2.77	7.43E-11
GPX3	7	4.27	16.95	2.32E-06
LOC781004	16	4.23	1.29	6.52E-05
LOC784738	26	4.23	2.74	0.0104
GALNTL2	1	4.16	7.00	6.42E-08
RASSF5	16	4.09	3.01	2.55E-05

UNC5B	28	4.09	6.90	2.00E-10
PEAR1	3	4.08	4.88	1.94E-08
ACER2	8	3.96	1.68	4.41E-08
KRBA1	4	3.94	2.30	4.61E-05
MGP	5	3.93	87.08	1.10E-06
ATP8B1	24	3.90	34.29	0.0298
DHX58	19	3.87	6.36	0.000676
LOC788414	2	3.86	2.81	5.41E-05
USP43	19	3.82	2.99	0.00126
IGFBP7	6	3.81	25.07	0.000558
GAS6	12	3.78	3.63	4.16E-08
LOC506672	2	3.72	2.72	0.0197
LOC535166	14	3.70	5.18	0.00313
LOC100337435	21	3.66	9.85	6.89E-07
BST2	7	3.65	41.38	0.00739
IFIH1	2	3.64	11.68	1.08E-05
CASQ2	3	3.60	1.61	0.00261
CCL26	25	3.57	1.83	0.0339
IGF2BP2	1	3.57	4.05	0.00158
OLFM2	7	3.53	6.46	0.000190
CAPS2	5	3.52	1.16	0.00671
ERG	1	3.51	1.34	0.00321
COL11A1	3	3.49	671.17	5.60E-06
BCAS1	13	3.48	5.53	0.00832
SEMA5B	1	3.48	19.25	2.60E-11
LOC100300816	25	3.48	1.01	0.000204
QPRT	25	3.45	1.03	0.0168
ARNTL2	5	3.44	3.12	0.00144
CACNA1A	7	3.40	25.47	0.000163
LOC100336535	19	3.36	1.09	3.84E-05
RASGRP2	29	3.30	4.91	0.00126
RARB	27	3.25	1.05	0.00507
SEMA5A	20	3.24	2.99	0.000723
USP18	5	3.24	8.54	0.000291
LOC789485	7	3.23	53.60	0.000211
MOCOS	24	3.22	9.66	0.00385
JAKMIP3	26	3.22	3.13	0.000197
ODZ4	29	3.17	3.93	0.000300
CMPK2	11	3.14	4.12	0.000472
ZMYND15	19	3.14	9.35	0.00292
COL8A1	1	3.12	9.31	0.0264
LOC100336868	16	3.10	1.89	0.00168
TGFB2	16	3.06	37.62	3.92E-07
PRUNE2	8	3.05	14.65	3.19E-08
RDH10	14	3.05	43.54	1.79E-07

WWC1	7	3.05	1.37	0.00834
CPM	5	3.01	26.63	0.00156
ITGA11	10	3.00	147.75	3.34E-06
ITPR3	23	3.00	1.53	0.00213
INHBA	4	2.98	35.87	4.54E-12
SPINT2	18	2.96	18.37	9.85E-11
LOXL3	11	2.95	16.15	3.56E-07
ICAM1	7	2.95	8.49	2.95E-05
LOC100847471	8	2.95	1.38	2.43E-05
RHOJ	10	2.95	17.62	3.68E-09
RAB11FIP1	27	2.93	2.79	0.00137
IGDCC4	10	2.92	2.67	0.00106
CCL2	19	2.91	159.46	1.47E-05
ARHGEF5	4	2.90	11.23	1.25E-05
CABLES1	24	2.90	12.43	2.09E-06
RTP4	1	2.90	2.79	0.000370
BDKRB1	21	2.89	7.56	0.00476
RASGRF2	7	2.88	3.25	0.00830
ITGA2	20	2.88	3.06	1.74E-05
KLHL32	9	2.87	3.92	1.05E-06
MARCKSL1	2	2.86	14.80	8.16E-06
PM20D1	16	2.85	7.07	0.000173
LPHN1	7	2.85	2.83	0.000113
C13H20orf112	13	2.84	1.88	0.00471
LOC100848128	11	2.84	2.20	0.0148
LOC100847896	15	2.83	2.65	0.0248
RHBDL2	3	2.83	2.69	0.00173
LOC782264	12	2.83	4.46	8.07E-05
WNT10B	5	2.81	2.25	0.000382
LOC100848019	9	2.77	67.42	0.0422
TNIP1	7	2.77	182.19	1.96E-06
XAF1	19	2.76	1.84	0.00429
ATP6AP1L	7	2.75	4.77	2.71E-05
LOC100847694	X	2.75	1.51	0.000617
SPEG	2	2.75	12.39	9.08E-09
LOC100847766	X	2.75	1.26	0.000902
LOC100847829	X	2.74	1.25	0.000928
LOC777601	X	2.74	1.25	0.000928
FAM83H	14	2.73	7.08	0.00343
IRF7	29	2.72	8.43	0.00486
SLC25A13	4	2.71	5.40	0.0233
C7H19orf66	7	2.71	2.37	8.42E-05
FAM115C	4	2.71	5.29	0.000860
SLC6A16	18	2.71	3.50	3.66E-06
BNC2	8	2.70	11.40	0.00486

GPR63	9	2.70	9.64	7.21E-05
AQP11	29	2.70	1.17	0.00188
RXFP4	3	2.70	1.50	0.000435
RNF213	19	2.69	105.73	1.54E-06
WDR35	11	2.68	37.99	2.49E-15
PROS1	1	2.66	104.56	0.00832
PIK3IP1	17	2.65	2.48	0.0215
TNFSF4	16	2.64	1.12	0.00900
LOC100299061	X	2.63	1.37	0.00114
LOC100141258	25	2.63	1.90	0.00829
MYCL1	3	2.63	1.07	0.000204
TNFAIP3	9	2.62	10.62	0.00176
LOC509006	7	2.61	60.57	2.25E-18
WNT2	4	2.60	3.53	0.00168
C8H9orf103	8	2.60	2.38	0.000382
ALDOC	19	2.59	15.65	0.00110
LOC100296463	25	2.59	11.78	4.66E-08
LOC615989	3	2.58	1.08	0.00813
CEACAM1	18	2.57	2.41	0.0439
IRF5	4	2.57	2.02	0.00370
PNPLA1	23	2.56	1.05	0.0323
SYN2	22	2.56	1.61	0.00732
CATSPERG	18	2.56	14.90	3.61E-13
GDAP2	3	2.56	15.17	0.00371
NKX2-2	13	2.55	2.02	0.00184
CES2	18	2.55	3.04	0.0301
HECTD2	26	2.54	5.32	9.63E-06
SPTBN4	18	2.54	1.09	0.00165
NFKBIA	21	2.53	57.84	2.06E-12
TLR6	6	2.52	7.53	8.07E-06
CYP7B1	14	2.52	8.72	0.00131
TP53I11	15	2.52	2.60	0.0440
LOC100848300	18	2.52	2.79	0.0422
ACSS2	13	2.52	2.88	0.0442
RGS9	19	2.52	4.07	0.0419
SEPT4	19	2.51	2.37	0.000361
TSPAN18	15	2.50	1.89	0.0175
TLR4	8	2.50	2.08	0.00547
LOC787074	18	2.50	1.14	0.0318
CSDC2	5	2.50	1.26	0.0120
MMP15	18	2.49	1.59	0.00893
SYNPO2	6	2.49	208.64	0.000259
DOK4	18	2.48	1.84	0.000586
CHRD	1	2.48	5.22	0.000394
VWF	5	2.47	1.86	0.00312

IRF9	10	2.46	15.57	0.00353
GDAP1	14	2.46	3.90	0.00741
MICALL2	25	2.46	7.56	1.54E-06
STX1A	25	2.45	1.64	0.0102
MARCH11	20	2.44	1.14	0.0162
GRAMD1B	15	2.44	3.53	0.000471
DDX58	8	2.43	15.46	0.00123
PKLR	3	2.43	1.13	0.00687
NMRAL1	25	2.42	7.46	0.00182
PRR5L	15	2.42	25.82	0.00266
SPON1	15	2.42	26.07	8.72E-07
FAM20A	19	2.41	18.31	0.000180
ENOX1	12	2.41	14.06	0.0367
HAVCR2	7	2.40	1.49	0.00354
LOC100847602	5	2.39	10.64	0.000630
KCNK6	18	2.39	10.85	5.57E-07
SORBS1	26	2.38	1.54	0.0472
TRPV4	17	2.38	2.84	0.00157
INPP1	2	2.38	12.84	3.24E-06
SDR42E1	18	2.37	1.01	0.00509
RHOD	29	2.37	4.30	0.0160
LOC784236	8	2.36	1.44	0.00186
HHIPL1	21	2.35	7.88	2.71E-05
EPSTI1	12	2.34	12.95	0.00400
LOC509420	8	2.34	1.22	0.00315
ITGBL1	12	2.34	16.64	0.000127
SPATA13	12	2.33	3.77	0.00322
CASP7	26	2.33	5.00	6.05E-05
SLC13A4	4	2.33	2.12	0.00886
ALOX12	19	2.33	3.09	0.00783
LOC100848191	18	2.33	84.37	2.31E-07
SHROOM3	6	2.32	10.41	0.000186
TSPAN12	4	2.31	2.89	0.0145
ATP8A2	12	2.31	9.61	2.72E-05
APBB2	6	2.31	57.08	6.57E-06
COL4A2	12	2.31	477.22	1.72E-08
LOC540363	17	2.31	1.79	0.00361
TNFRSF25	16	2.30	2.68	0.00180
JAG1	13	2.30	215.06	0.000124
UBA7	22	2.29	21.73	0.00102
CXCL16	19	2.29	54.35	0.00763
HSPB6	18	2.28	98.40	6.42E-08
CPXM2	26	2.28	1.67	0.0301
LOC100848206	X	2.27	3.59	0.00252
LOC789525	18	2.27	1.24	0.00239

ANKH	20	2.27	76.12	2.00E-10
LOC100336734	18	2.26	1.51	0.0196
EFEMP1	11	2.26	61.39	0.00547
LMTK3	18	2.26	2.57	0.00346
LOC539821	16	2.26	1.42	0.00323
DMPK	18	2.24	6.26	7.71E-06
SDC1	11	2.24	79.84	9.70E-07
GPR17	2	2.24	1.24	0.0297
IFITM1	29	2.23	6.91	0.0162
P4HA3	15	2.23	9.68	0.00171
CLDN11	1	2.23	1.53	0.0353
GREB1L	24	2.23	10.65	0.00207
MSC	14	2.23	7.27	0.000139
C5H12orf66	5	2.23	1.39	0.00266
LOC100848263	19	2.23	6.97	2.80E-05
FN1	2	2.23	11609.64	0.000617
DTNA	24	2.21	4.05	0.00847
LOC100848597	25	2.21	1.52	0.00323
PTHLH	5	2.21	9.28	0.00586
GUCY1B3	17	2.21	39.93	5.77E-07
ABCA3	25	2.21	9.43	0.0294
LAMC2	16	2.20	6.05	0.0106
TCF7	7	2.20	12.03	0.000572
IGFBP3	4	2.20	65.93	0.00385
ABCA4	3	2.20	2.23	0.000917
UNC5C	6	2.20	5.40	0.0365
RNF125	24	2.19	5.45	0.000957
ALDH1A3	21	2.19	117.04	0.0156
RARRES2	4	2.19	7.21	0.0127
PLTP	13	2.18	3.94	0.00323
LOC525353	26	2.18	3.71	0.0169
ZSWIM4	7	2.18	11.73	2.93E-07
BIRC3	15	2.17	49.76	0.000199
LOC100849059	7	2.17	3.04	0.0176
PARP12	4	2.17	18.84	2.21E-05
FES	21	2.17	6.31	0.0371
C12H13orf15	12	2.17	62.41	7.27E-05
SCG5	10	2.17	7.44	1.12E-05
MRVI1	15	2.17	14.14	0.000178
ENC1	20	2.17	138.63	1.98E-06
COL4A1	12	2.17	1620.80	7.90E-06
MGC148692	6	2.16	1.03	0.00941
CASQ1	3	2.16	3.77	0.0182
CPT1C	18	2.15	1.54	0.0246
TRANK1	22	2.15	24.61	4.25E-05

IFITM2	11	2.14	16.03	0.0131
C3	7	2.14	6.41	0.000541
LOC100848886	21	2.14	1.83	0.00238
ANKRD23	11	2.14	1.54	0.0297
MANSC1	5	2.13	5.73	0.0131
MMP28	19	2.13	4.25	0.000260
CYP3A5	25	2.13	129.68	0.0205
BCAM	18	2.13	9.98	2.97E-05
OLFML2B	3	2.13	51.52	0.0148
KCNC4	3	2.12	1.28	0.0110
AK5	3	2.12	1.23	0.0288
AGMO	4	2.12	25.30	1.77E-11
LOC100848842	19	2.10	4.03	0.00683
OBSL1	2	2.10	11.39	0.00883
DOCK10	2	2.09	35.62	3.84E-05
PARP9	1	2.09	15.02	0.000272
CDC42EP2	29	2.09	1.91	0.0104
HSF4	18	2.08	5.06	0.0380
AGPAT4	9	2.07	9.85	0.00125
SARM1	19	2.07	4.13	0.0180
CHPF	2	2.07	121.62	3.87E-05
PPAP2B	3	2.07	18.72	0.0115
LOC100847429	13	2.07	25.19	0.000119
TNFAIP6	2	2.05	10.17	0.00154
CTSZ	13	2.05	68.02	3.14E-06
GLIPR1	5	2.05	7.36	0.00771
CD40	13	2.04	36.47	8.42E-05
COL5A3	7	2.04	179.91	0.000381
MOSC2	16	2.04	3.69	0.00442
MAGI1	22	2.04	20.16	0.000366
IL34	18	2.04	11.73	0.000608
LIMS2	2	2.03	7.06	0.0379
CHCHD6	22	2.03	1.44	0.00595
ASAP3	2	2.02	8.61	0.0208
LOC786974	20	2.02	8.62	1.93E-05
TPD52	14	2.02	8.72	0.00112
FADS6	19	2.02	3.53	0.0355
CASK	X	2.01	86.72	5.01E-05
ZFHX4	14	2.01	13.23	0.0193
LOC100336905	X	2.01	4.35	0.00428
CYBA	18	2.00	21.79	0.000844
FILIP1	9	2.00	9.37	0.000734
TRIM21	15	2.00	6.78	0.000133
ARHGAP29	3	2.00	131.36	6.16E-08
EXO1	16	-2.00	7.71	0.00287

CDKN3	10	-2.00	19.70	0.00140
CDC20	3	-2.01	68.24	0.000352
C1QTNF2	7	-2.01	1.42	0.0295
OIP5	10	-2.02	12.79	0.00240
LOC615206	26	-2.02	2.99	0.0141
LOC100848433	4	-2.03	103.02	0.000135
LOC784206	4	-2.03	1.14	0.0231
ARL4A	4	-2.03	1.12	0.0225
KIAA0101	10	-2.04	30.59	0.000266
RRM2	11	-2.05	203.81	0.000287
CEP72	20	-2.06	11.01	0.00323
PTGR1	8	-2.06	16.52	0.00997
SAMD11	16	-2.06	14.70	0.000164
CCNB2	10	-2.06	28.64	0.000231
LOC100848655	5	-2.07	17.20	0.000272
TNFRSF6B	13	-2.08	18.85	0.0214
MRGPRF	29	-2.08	40.50	4.50E-06
KIF20A	7	-2.09	123.98	0.000282
CDCA4	21	-2.09	4.63	0.0373
LOC100299874	9	-2.09	7.78	0.00317
FOSL1	29	-2.10	65.20	2.84E-08
CDCA3	5	-2.10	32.49	0.000497
STMN1	2	-2.10	72.57	0.000119
MYBL2	13	-2.10	85.76	0.000146
NFIB	8	-2.10	1.64	0.00708
CCDC34	15	-2.10	11.62	0.000179
PMCH	5	-2.12	3.99	0.00560
GPR176	10	-2.12	1.07	0.00861
TSPAN13	4	-2.12	59.58	9.85E-11
NXT1	13	-2.13	10.28	7.52E-05
PEG10	4	-2.13	8.07	0.000819
PKD2L1	26	-2.15	11.02	0.000522
LOC513969	13	-2.16	2.10	0.00296
TOP2A	19	-2.16	277.71	0.000139
IRX1	20	-2.17	5.66	8.41E-05
CENPN	18	-2.19	18.39	7.24E-05
ASPM	16	-2.20	103.55	2.63E-05
MAD2L1	6	-2.20	36.35	3.84E-05
LOC782456	13	-2.20	1.24	0.00925
FABP3	2	-2.21	9.99	4.03E-05
ROBO2	1	-2.22	5.32	0.00948
CHST1	15	-2.22	5.98	0.000434
DUSP5	26	-2.22	1.56	0.0188
ZNF608	7	-2.23	1.32	0.0189
CADM3	3	-2.23	2.31	0.00526

EPHB4	25	-2.24	33.09	1.73E-07
HSPA12A	26	-2.25	42.62	1.08E-06
GAS2L3	5	-2.27	13.42	0.000329
TFAP4	25	-2.28	2.39	0.0406
PCOLCE2	1	-2.28	8.49	0.00196
E2F8	29	-2.28	14.95	0.000163
ZAN	25	-2.29	4.19	0.000143
NFATC2	13	-2.29	1.29	0.0169
TMEM158	22	-2.29	21.50	4.61E-05
CCDC85B	29	-2.29	19.43	0.000157
LOC100297402	4	-2.29	1.33	0.00600
EPHB2	2	-2.29	3.54	0.0365
HMOX1	5	-2.29	20.10	1.64E-08
LOC783804	26	-2.29	7.68	0.000190
LOC100848911	7	-2.29	31.82	2.96E-05
LOC100336768	9	-2.30	1.02	0.00749
LOC782601	27	-2.31	4.50	0.00323
PM20D2	9	-2.33	2.37	0.00160
GREM1	10	-2.34	87.33	6.51E-06
SOCS1	25	-2.35	1.24	0.0165
GJB3	3	-2.37	1.33	0.00914
ID1	13	-2.38	45.31	2.84E-08
CENPA	11	-2.38	44.10	5.38E-05
KIF26B	16	-2.41	25.94	0.0137
LRRN3	4	-2.41	8.38	0.00143
HSPB8	17	-2.41	40.05	1.72E-06
LOC100848808	4	-2.44	13.68	1.00E-07
ADM	15	-2.45	44.79	1.74E-08
FAM13C	28	-2.46	12.71	0.00649
CYP26B1	11	-2.47	21.58	4.28E-05
HS3ST2	25	-2.49	65.28	5.12E-05
AKAP6	21	-2.50	24.54	0.000164
AHRR	20	-2.50	1.67	0.00677
PIR	X	-2.50	6.26	0.000162
SPOCK1	7	-2.53	12.02	0.00264
B3GALNT1	1	-2.57	11.04	1.59E-05
PDE10A	9	-2.60	5.00	0.000498
MAD2	6	-2.63	36.34	0.0202
MAN1C1	2	-2.63	9.09	0.000819
LOC100848949	1	-2.66	4.87	0.000369
SP7	5	-2.66	1.81	0.00146
ANKRD6	9	-2.67	5.32	0.00274
LOC529036	1	-2.69	1.40	0.00431
CSMD2	3	-2.70	2.86	0.00121
RPL7A	11	-2.73	550.79	0.0208

KCNN4	18	-2.73	1.55	0.0173
HPGD	8	-2.74	26.03	5.83E-05
MESP2	21	-2.76	1.08	0.00170
LOC100847220	X	-2.78	1.15	0.00305
EFNB2	12	-2.79	2.19	0.000190
E2F2	2	-2.80	1.20	0.00169
PDE1C	4	-2.80	3.40	0.00394
PPL	25	-2.82	10.57	6.20E-12
GDA	8	-2.83	1.65	1.79E-05
PCDH11Y	X	-2.85	60.37	0.000522
C11H2orf40	11	-2.86	2.67	0.0197
PIF1	10	-2.88	1.15	0.0100
GLI1	5	-2.89	1.86	0.00131
LOC781565	1	-2.91	18.27	0.0334
ADRB2	7	-2.98	8.40	4.99E-06
AQP1	4	-2.98	487.82	1.03E-06
HDAC9	4	-2.99	4.49	0.000439
MMP27	15	-3.00	2.61	0.00293
CTSL1	8	-3.04	3.11	0.00189
GSG2	19	-3.10	16.48	0.000231
MEOX1	19	-3.16	1.40	8.58E-05
LOC510913	24	-3.20	2.05	0.0154
PCDH17	12	-3.21	1.90	0.0362
XPNPEP2	X	-3.23	16.16	4.51E-10
CTSW	29	-3.23	2.19	1.28E-06
SYT17	25	-3.27	1.05	0.000204
LBH	11	-3.27	9.66	5.41E-05
LRRN4CL	29	-3.36	105.14	5.43E-13
ADAMTSL1	8	-3.41	8.40	9.85E-11
PTGS1	11	-3.45	45.01	1.10E-06
SCN9A	2	-3.54	3.87	2.31E-05
SOD3	6	-3.54	2.68	0.00144
THBD	13	-3.55	24.92	2.71E-14
MAP2	2	-3.65	2.50	7.36E-05
EFCAB4B	5	-3.67	1.07	7.06E-06
ESM1	20	-3.69	73.70	1.80E-06
LOC100847727	4	-3.75	13.91	1.47E-19
RAVER2	3	-3.89	1.39	6.61E-05
COL6A6	1	-3.89	3.03	0.0323
EMID1	17	-3.92	1.53	0.000572
EFNA5	7	-4.01	1.12	0.000133
COL13A1	28	-4.01	1.23	0.00118
PPP1R14C	9	-4.07	1.11	0.00186
SCN1A	2	-4.07	3.06	3.96E-06
NGEF	3	-4.07	2.03	0.0134

DKK2	6	-4.08	7.33	1.62E-06
LPXN	15	-4.09	1.32	1.33E-07
FRMD4B	22	-4.12	1.63	0.00510
LOC100139916	25	-4.12	1.27	0.00102
LOC509513	4	-4.13	1.15	0.0207
CXHXorf57	X	-4.23	8.89	6.47E-06
ARHGAP18	9	-4.34	12.13	6.18E-09
RXFP1	17	-4.51	1.30	0.000545
NDP	X	-4.55	1.17	1.51E-05
COLEC12	24	-4.59	6.57	3.10E-09
ADAMTSL1	8	-5.25	2.04	0.000126
LOC100300510	4	-5.28	1.02	0.00714
RPS27	17	-5.30	14.65	0.0318
MEGF6	16	-5.85	63.11	5.09E-12
F3	3	-6.15	77.22	2.53E-14
CXCL12	28	-6.45	25.55	6.87E-07
FBP1	8	-6.71	1.54	2.32E-10
NLGN4Y	X	-7.39	2.90	1.58E-08
LOC100848103	25	-7.81	9.56	1.20E-28
LOC515128	16	-7.82	1.19	0.00125
CRISPLD1	14	-9.06	5.17	0.00131
ANKRD24	7	-9.23	3.88	1.99E-13
KCNG1	13	-9.56	1.67	2.79E-07
ASIP	13	-13.42	17.58	6.97E-34
CYP27C1	2	-15.10	1.56	1.34E-07
SLC32A1	13	-19.16	1.41	3.19E-13
COBL	4	-21.12	1.48	2.82E-05
LOC100296277	X	-39.74	7.39	0.00172
RBM44	3	-137.35	5.54	1.80E-05
PRSS2	4	-3892.84	3.10	2.93E-06
